# Public voices on tie-breaking criteria and underlying values in COVID-19 triage protocols to access critical care: a scoping review

**DOI:** 10.1007/s44250-023-00027-9

**Published:** 2023-05-10

**Authors:** Claudia Calderon Ramirez, Yanick Farmer, Marie-Eve Bouthillier

**Affiliations:** 1 Biomedical Sciences Program, Clinical Ethics, Faculty of Medicine, Université de Montréal, 2900 Bd Édouard-Montpetit, Montréal, Québec H3T 1J4 Canada; 2 Department of Social and Public Communication, Faculty of Communication, Université du Québec à Montréal, C.P 8888, Succursale Centre-Ville, Montréal, Québec H3C 3P8 Canada; 3 Department of Family and Emergency Medicine and Office of Clinical Ethics, Faculty of Medicine, Université de Montréal, 2900 Bd Édouard-Montpetit, Montréal, Québec H3T 1J4 Canada

**Keywords:** COVID-19 triage, Tiebreakers, Public consultation, Clinical ethics, Scoping review

## Abstract

**Background:**

To reduce the arbitrariness in the allocation of rare resources in intensive care units (ICU) in the context of the pandemic, tiebreakers were considered in some COVID-19 triage algorithms. They were also contemplated to facilitate the tragic decisions of healthcare workers when faced with two patients with similar prognosis and only one ICU bed available. Little is known about the public's perspective on tiebreakers.

**Objectives:**

To consolidate the available scientific literature on public consultations, particularly on tiebreakers and their underlying values. Also, to obtain an overview of the key arguments presented by the participating public and to identify potential gaps related to this topic.

**Methods:**

The steps described by Arksey and O’Malley was the preferred method to our approach. Seven electronic databases were searched from January 2020 to April 2022, using keywords for each database: PubMed, Medline, EMBASE, Web of Science, PsycINFO, EBM reviews, CINAHL complete. We also searched in Google and Google Scholar, and in the references of the articles found. Our analysis was mainly qualitative. A thematic analysis was performed to consider the public’s perspectives on tiebreakers and their underlying values, according to these studies.

**Results:**

Of 477 publications found, 20 were selected. They carried out public consultations through various methods: surveys (80%), interviews (20%), deliberative processes (15%) and others (5%) in various countries: Australia, Brazil, Canada, China, France, Germany, India, Iran, Italy, Japan, Korea, Netherlands, Portugal, Spain, Switzerland, Thailand, United Kingdom, and United States. Five themes emerged from our analysis. The public favored the life cycle (50%) and absolute age (45%) as a tiebreaker. Other values considered important were reciprocity, solidarity, equality, instrumental value, patient merit, efficiency, and stewardship. Among the new findings were a preference for patient nationality and those affected by COVID-19.

**Conclusions:**

There is a preference for favoring younger patients over older patients when there is a tie between similar patients, with a slight tendency to favor intergenerational equity. Variability was found in the public’s perspectives on tiebreakers and their values. This variability was related to socio-cultural and religious factors. More studies are needed to understand the public's perspective on tiebreakers.

**Supplementary Information:**

The online version contains supplementary material available at 10.1007/s44250-023-00027-9.

## Background

COVID-19 has caused repercussions of varying magnitude throughout the world. This viral disease with an accelerated epidemiological behavior, was declared a pandemic by the WHO in March 2020, less than 3 months after its appearance in China [1]. Its most severe clinical manifestation was in the lungs, which led to an increased demand for invasive ventilators and intensive care unit (ICU) beds around the world [2, 3]. Triage as a strategy for prioritizing care when available health resources are scarce has been promoted by various experts [4–9]. Health authorities in various countries have had to develop triage protocols to deal with the potential shortage of these limited health resources. The emphasis on triage protocols for access to ICU beds in this pandemic has been remarkable. However, many protocols developed in various countries have not been published. Thus, some of them will change in structure as this pandemic unfolds and scientific knowledge develops. The evolution of triage protocols is proving to be a dynamic process [4, 5, 7].

Triage protocols in a pandemic context have been strategies that have raised ethical issues related to their values and criteria for the allocation of scarce health resources [9–12]. Among the fundamental ethical principles for prioritizing care in this pandemic situation, maximizing benefits and treating patients equally stand out. However, a single basic value is not enough to determine the rationing of a resource for one patient over another. Therefore, it is necessary to have an approach that contains several values to make this prioritization [4, 6, 9].

In the literature, there are reviews of COVID-19 and other pandemic protocols for ICU access. We found systematic reviews on the allocation of health resources during the influenza pandemic and disaster situations, some containing public consultations [13–16]. There were also recent literature reviews related to the COVID-19 pandemic and prioritization protocols [17–23]. For example, a systematic review found 83 published protocols for the COVID-19 pandemic, as well as other pandemics. They conducted a review of the criteria and values considered in these protocols, finding only 30 studies reporting the principles and values underlying their admission criteria, and of these, only 16 COVID-19-related studies described them [17].

It is important to note that some protocols for ICU access include both initial clinical criteria and tiebreaker criteria, the latter considered by some to be non-clinical, with underlying values [16, 17, 19, 23, 24]. Initial clinical criteria are core criteria that are used at the first point of contact for patients requiring admission to the ICU. These core criteria are generally considered worldwide. For example, they consider the urgency and severity of the patient's illness, and the vital prognosis such as acute and chronic comorbidities or the probability of death for poor prognosis diseases or degenerative diseases, among other aspects. These primary prioritization criteria can be based on both standardized clinical scales or scores, without missing the clinical judgment of the physicians or the triage team [23–25]. In contrast, criteria considered tiebreakers or supplementary or non-clinical criteria, have been developed for secondary use in ICU admission decisions. They can be used to resolve an impasse when purely clinical criteria are no longer sufficient to prioritize patients in the same clinical situation. These include absolute age, life cycle, social or instrumental value, multiplier effect, or randomization, among others. They could be applied in the event of extreme resource shortages in the ICU, a situation in which only one available space must be allocated [16, 19, 26].

A systematic review compiled the criteria of some ventilator allocation guidelines developed in the USA for access to adult intensive care in the face of this pandemic. The literature showed that after considering their initial prioritization criteria, if there was a tie between patients, they would consider the younger patients as an initial tiebreaker, in 6 states (Pennsylvania, Oklahoma, Michigan, Massachusetts, Colorado and South Carolina). In 2 states, they would also consider healthcare workers, who are the most exposed (Colorado and Oklahoma). In 5 states, they would consider the “first-come, first-served” principle as a tiebreaker (Washington, Michigan, Kansas, Indiana, and Maryland) [19]. However, we do not currently know the total number of COVID-19 protocols already developed with tiebreakers included in their criteria.

During the influenza pandemic, protocols were also developed to address a shortage of resources, and tie-breaking criteria were included in their structure. Some communities were consulted on these criteria and values, showing a diversity of perspectives [13, 14, 27–31].

For example, a recent systematic review compiled 24 studies of other pandemics between 2004 and 2019 in which various methods of community engagement (deliberative processes, focus groups, and/or interviews) were applied to include the public in the evaluation of pandemic planning and its response. Participants' perspectives were also analyzed on these health strategies. They also found diverse and divergent perspectives among community participants and a consensus was difficult to obtain [32].

Public engagement in the development and approval of health protocols is proving to be an important element in the search for its legitimacy [33–35]. The opinion of the public in considering these tiebreakers in the structure of the COVID-19 protocols could not be obtained in most cases due to the urgency of their elaboration, most of which were planned during the first wave of the pandemic. Most consultations were carried out later, for reasons of transparency and to seek the legitimacy of these health strategies. Being aware of this situation, we do not expect to find much literature on this subject.

The purpose of this scoping review was to: consolidate the available scientific literature on public consultations conducted as part of the COVID-19 pandemic, particularly on tie-breaking criteria and the underlying values of prioritization protocols for ICU access, as well as providing a snapshot of current knowledge, including an overview of the key arguments of public perceptions, and identifying potential gaps related to this topic.

## Methods

The steps described by Arksey and O’Malley (2005) was the preferred method, as it allowed us to adapt our approach with flexibility [36, 37]. Initial ethics approval was not required for this study, as it was documentary research, not directly research involving human subjects.

### Identifying the research question and the rationale of the design

There are few studies of public opinion regarding the COVID-19 protocols for access to critical care and the primary and secondary criteria they contain. There is also little knowledge about the public arguments surrounding these criteria. We considered it important to learn more about these tiebreaker criteria and the perspectives the public has on them. Therefore, our main research question in this review was: *What are the public’s views on the tiebreaker criteria contained in the COVID-19 prioritization protocols?* To obtain the answer to this question, we considered it pertinent to carry out a scoping review.

Our scoping review involves the Arksey and O’Malley methodological design because of its flexibility in the analysis of empirical and normative literature, as well as the possibility to perform a quantitative and qualitative analysis at the same time. This design appears to be a valuable option for research in bioethics and clinical ethics [38]. However, according to our objectives based on the main question and the search for arguments in relation to ethical issues, we decided to focus this scoping review on a qualitative analysis.

### Eligibility criteria

The inclusion criteria were:

- Studies based on public consultations of COVID-19 triage protocols developed for adult patients for access to the ICU describing the tie-breaking criteria and underlying values.
- Studies based on public consultations whose topic was related to tiebreakers and/or underlying values considered in prioritizing resources for adults in the ICU during this pandemic (not including protocols developed for this purpose).
- Studies that have undergone public consultations through a variety of methods: surveys, interviews, focus groups, deliberative or mixed processes.
- Studies with quantitative, qualitative, or mixed methodology specifications published from January 2020 to April 2022.
- Studies that have not yet been published or that were not peer-reviewed were excluded.

### Search strategy

The collaboration of a librarian was sought for the search strategy and for obtaining articles in English, French and Spanish. Seven electronic databases were searched using keywords, with adaptations made based on the controlled vocabulary for each database: PubMed, Medline, EMBASE, Web of Science, PsycINFO, EBM reviews, CINAHL complete. We also searched the grey literature (Google Search and Google Scholar) and performed a scrutiny on the references of the articles found. Initial search terms included: COVID-19 triage, Intensive Care Unit, tiebreakers, life cycle, social utility, biological age, ethical values, surveys, deliberative processes, interviews, and others (Related File 1: Database Search Strategies).

### Identifying relevant studies

For the database search strategy, the first search was carried out in February 2022. To obtain more studies that would offer us more data and perspectives from the public, the PubMed database was launched for a second and third search in April 2022. In this third search, it was necessary to modify the controlled vocabulary to obtain more articles related to our research subject, and we obtained more interesting articles. Through the manual search in Google and Google Scholar, we also found some interesting preprint articles already accepted for publication, but not yet published so it was not possible to include them in our results. We also thoroughly searched the reference section of the selected articles from which we obtained other interesting articles.

### Study selection

The eligibility criteria for the search of articles from each database were initially applied by an independent person and one of the authors (CC). The manual search of articles was performed by (CC) both in Google and Google Scholar, as well as in the references section contained in the selected articles. One author (CC) initially selected the titles and abstracts relevant to the research subject obtained from the databases using EndNoteX9©, eliminated duplicates, and appended those from the manual search. The full-text articles were then reviewed and verified according to the eligibility criteria by two authors independently (MEB) and (YF) to resolve discrepancies and reach a consensus.

### Charting the data

**Fig. 1:**
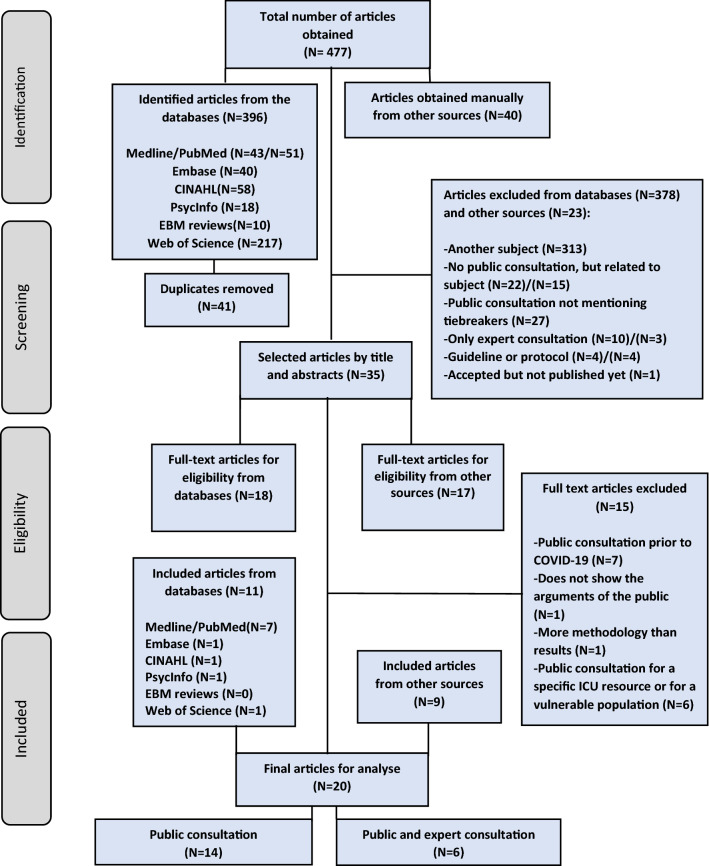
PRISMA flow diagram of selected articles for thematic analysis

A flow chart was prepared according to the PRISMA Extension for scoping review (PRISMA-ScR)^©^ to present empirical papers [39] (Fig. 1).

**Fig. 2:**
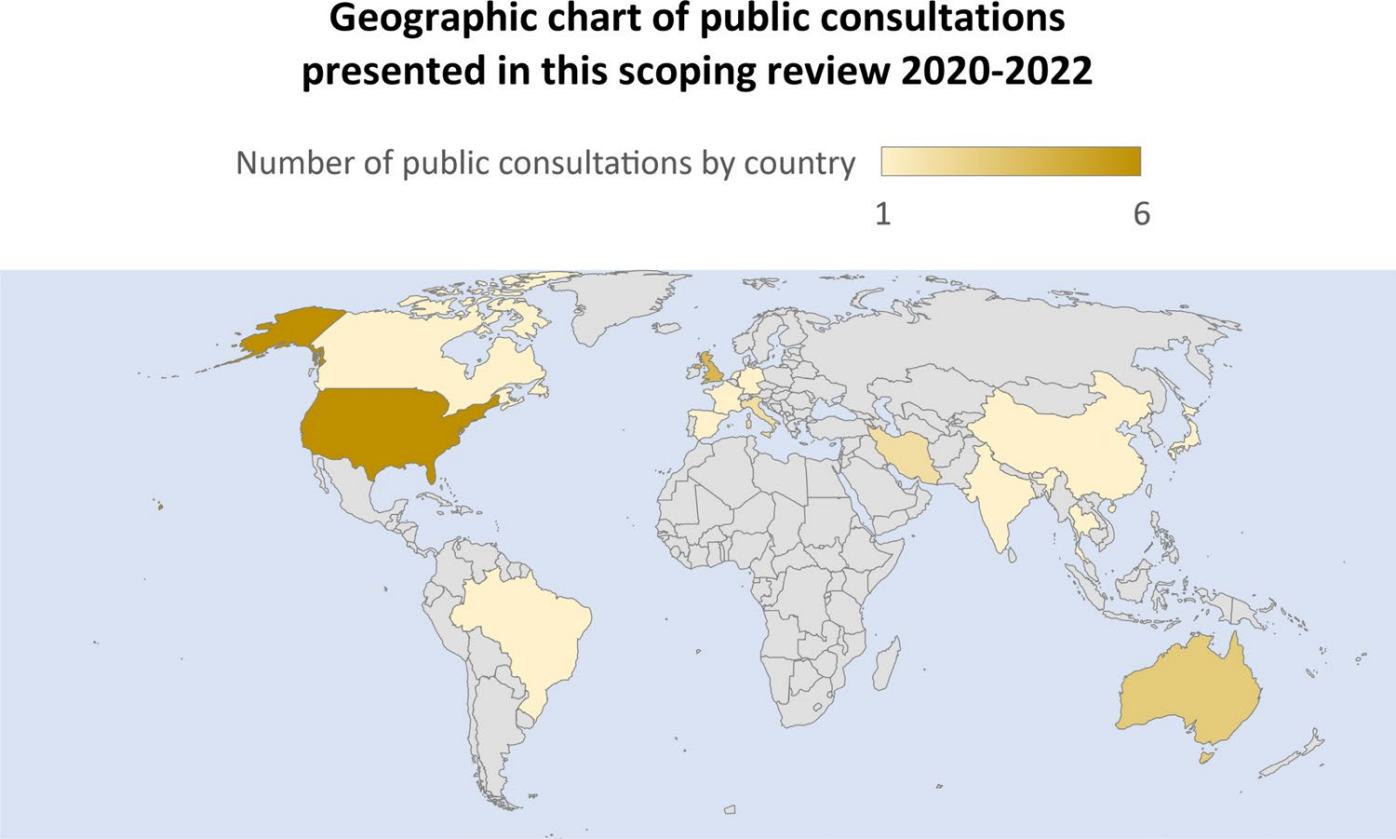
Geographic chart of public consultations by country. Source: ©Australian Bureau of statistics, GeoNames, Geospatial Data Edit, Microsoft

Tables and graphs were prepared by one author (CC) and checked by two authors (MEB) and (YF) to assess the accuracy of their content. Descriptive statistics were used for the data in percentages. Excel© software was used for quantitative data management. The tables show the main characteristics of the articles found and the most relevant arguments of the public (Author, year, country, population consulted, type of intervention/methodology) (Table 1) and (Arguments for and against each of the tiebreakers) (Table 2). We also present a geographic chart of the countries that have made public consultation according to this review (Fig. 2).

**Table 1:** Main characteristics of public consultations on tiebreakers for ICU access in a pandemic context N = 20

N^o^	Author	Year	Country	Type of intervention/Methods	Population		References
					(N =) Total of participants/(%) of public	Target group	
1	Abbasi et al	2021	Iran	Online survey/Quan	N = 1769 (87)	Public/HCW	[49]
2	Asghari et al	2021	Iran	Online survey/Quan	N = 767 (68.8)	Public/HCW	[50]
3	Bruno et al	2021	USA	Online survey, interviews and deliberations/Quan and Qual	N = 21 (33.3)	Public/experts	[42]
4	Chan et al	2022	UK	Two online surveys/Quan	N = 525 (100)	Public	[46]
5	Falluchi et al	2021	USA	Online survey/Quan	N = 1033 (100)	Public	[43]
6	Géa et al	2022	USA/Canada	Online survey/Quan	N = 583 (100)	Public	[41]
7	Gisjbers et al	2021	Netherlands	Online survey/Quan	N = 243 (100)	Public	[55]
8	Huseynov et al	2020	USA	Online survey/Quan	N = 586 (100)	Public	[44]
9	Jin et al	2021	USA, Brazil, India, UK, Italy, Germany, France, Australia, Spain, China, and South Korea	Online survey/Quan	N = 5175 (100)	Public	[40]
10	Knotz et al	2021	Switzerland	Two online surveys/Quan	N = 1450/1457 (100)	Public	[54]
11	Kuylen et al	2021	UK	Online deliberations/Qual	N = 22 (100)	Public	[47]
12	Lee et al	2021	Korea	Online survey/Quan	N = 1509 (100)	Public	[59]
13	Marshall et al	2021	Thailand	SR, interviews (only experts) and deliberations: 9 online and 2 in person (experts/public)	N = 21 (52.3)	Public/experts	[57]
14	Norman et al	2021	Australia	Online survey/Quan	N = 1050 (100)	Public	[51]
15	Norisue et al	2021	Japan	Online survey/Quan and Qual	N = 1520(43.8)	Public/HCW	[52]
16	Pinho et al	2021	Portugal	Online survey/Quan	N = 586 (100)	Public	[56]
17	Rai et al	2021	USA	Online survey/Quan and randomized trial	N = 700 (100)	Public	[45]
18	Riccioni et al	2021	Italy	Comments by e-mail and website	N = 16 (50)	Public/experts	[53]
19	Street et al	2021	Australia	Online survey/Quan	N = 306 (100)	Public	[52]
20	Wilkinson et al	2020	UK	Online survey/Quan	N = 763 (100)	Public	[48]

*ICU*   intensive care unit, *HCW*   health care workers, *Quan * quantitative analysis, *Qual*   qualitative analysis

*UK*  United Kingdom, *USA*  United States of America, *SR*   systematic review

**Table 2:** Tiebreakers for accessing intensive care in the context of pandemics and the main public arguments

Tiebreakers considered in public consultations	Discussion of criteria (%)	Main public arguments in favor	Main public unfavored arguments	Citations
1. Age-related priority Life cycle Absolute age “Saving more years of life” Principle	90	• Highly favor pregnant women [49] • Favor the youngest over the oldest considering the life cycle [40, 44, 46, 51, 52, 55, 56, 58, 59] • Reveal their support for the youngest independent of their prognosis [56] • Favor the youngest, considering age relevant [40, 41, 48, 49, 52, 53, 56, 59] • They indicated that prognosis depends on age [56] • They associated age with prognosis, life expectancy and quality of life [46] • They stated that outcomes under intensive care may be worse in older age [53] • The principle of ‘‘saving more years of life’’ ^a^is preferred as a tiebreaker because it excludes the elderly and those with severe comorbidity [45]	• They do not find justification between a patient 20 years older than the other, and consider the life cycle unfair [47, 50] • They do not consider the patient’s age as a prioritization criterion [54] • In the deliberations, they rejected the absolute age criterion, even in the form of “number of years of life saved”^a^, because of the risk of discriminating against the elderly [57]	^a^It was a poorly defined principle, varied in meaning [90]. For participants apparently it would be to ensure a long-term life after intensive care intervention, in (Rai et al. pages 4–6) and (Marshall et al. page 8)
2. Priority for healthcare personnel Gratitude Multiplier effect	60	• Consensus in favor of the most exposed healthcare personnel, they will be prioritized in gratitude (reciprocity) but not the same for the non-exposed [41, 42, 46, 48, 50, 58] • They favor the most exposed health personnel for their multiplier effect (instrumental value) [42, 43, 46, 48, 49, 56] •* “If you really want to curb the spread and impact of the virus, you need to prioritize those who can help others and save more lives.”* ^b^ [42] • They gave the highest priority to essential healthcare workers [41] They favor the prioritization of essential healthcare workers, but their values were not defined [51, 52]	• For some, the perspective of the instrumental value of health personnel was arbitrary and impractical and was discarded [57]	^b^A participant indicated this in a deliberative process, emphasizing the importance of prioritizing essential healthcare workers, in (Bruno et al. page 394)
3. Priority to essential people Parent caregivers Caregivers of vulnerable people	40	• They favor mothers in charge of minor children and people with dependents [46, 48, 49, 51, 52, 58]	• They reject the idea of prioritizing according to the social value of a person by considering life of equal value [57] • They disagreed as equal opportunity should be given to patients without dependents [50]	
4. Priority based on equality “First come, first served” Principle Randomization (lottery or draw)	40	• They preferred the “first-come, first-served” principle to randomization as a tiebreaker [43, 47, 59] • Favor randomization as a tiebreaker of last resort, after considering age and healthcare workers [48] • *That’s where I see the lottery come in. We should treat everyone equally at that point”* ^d^ [42]	•* “Lottery doesn’t feel like a good system when it’s people lives. It’s inappropriate”* ^c^ [47] • *First come, first served isn’t egalitarian and you have the socio-economic challenge..*”^e^ [47] • Strongly disagree with the “first-come, first-served” principle as they consider it unfair [48, 58] • Disfavor both the “first-come, first-served” principle and randomization, and consider them irrelevant [52, 55]	^c^A participant indicated disagreement in using the lottery, perhaps in the sense that we cannot gamble with life and that a suitable method should be sought, in (Kuylen et al. page 293) ^d^While another participant may have considered the lottery to be the fairest form of tiebreaker, in (Bruno et al. page 396) ^e^ A participant expressed disagreement by considering that the “first-come, first served” principle was not entirely fair, referring to the socio-economic situation of the patient, since it could disadvantage those who are not socio-economically well off, in (Kuylen et al. page 293)
5. Merit-based priority Volunteer background Responsible health conduct First responders (police, firemen and others) Criminal or social misconduct background	40	• They favor the benevolent and well-behaved patients before and during their COVID-19 infection [54, 55] • They favor prioritization based on the merit of people considered to be first responders [42, 43]	• They disfavor patients with criminal records (violent crimes), social irresponsibility, and for their high-risk activities, including smoking [40, 41, 46, 51]	
6. Priority by vulnerability Neurodevelopmental or psychiatric background COVID-19 and non-COVID-19 patient Fragility Physical disability	20	• They favor vulnerable people with COVID-19, indicating the avoidance of giving them less care because they are infected [41] • Favor COVID-19 versus non-COVID-19 patients in case of equality [43] • Favor in case of similarity the less frail patient either with handicap or learning disability [48]	• They disadvantage patients with psychiatric problems and medical-legal background in the face of a tiebreaker [41] • Disadvantage patients with physical, neurodevelopmental, and mental handicaps in the face of a tiebreaker [51]	
7. Priority by patient's nationality	20	• They favor both foreigners and nationals on an equal footing (no distinction) [49]	• Disfavor foreigners in case of similar prognosis between patients, favoring those of their nationality [40, 54, 59]	
8. Priority according to the patient's quality of life	15	• They favor the vulnerable patient but with good quality of life, suggesting this criterion as a tiebreaker [46, 47]	•* “I don’t know if professionals can really confirm how somebody’s wellbeing is”* ^f^ [47] • They refuse to evaluate the patient's quality of life because of its subjectivity [50] • They consider that the patient's age is important because it can influence the quality of life [46]	^f^One participant may have considered that this assessment of quality of life is not realistic to what each person perceives as quality of life, in (Kuylen 2020 page 293)
9. Priority based on rapid recovery of the intensive care patient	15	• They favor patients who need short periods of intensive care treatment (short-term recovery) in case of tiebreaker [47, 48, 58]		
10. Priority based on financial and human cost	5	• Favor patients incurring lower health care costs (e.g., less time spent on a ventilator in intensive care) [40]	• They disfavor irresponsible people by infecting others, increasing the risk of human losses [40]

### Process of thematic analysis and data synthesis

Following a coding structure developed by the team members, one author (CC) extracted the most salient arguments from the public consultations and the results found from the selected studies. All authors coded separately these segments and the participants' views that were considered most relevant. These arguments allowed the emergence of themes and sub-themes related to the tiebreakers considered by the public in the studies found. After discussing the themes, a consensus was reached among authors. In general, a synthesis of qualitative data was made (Related File 2: Qualitative synthesis).

## Results

This scoping review aimed primarily to identify the arguments of the public captured by different methods of public consultation in relation to the prioritization in intensive care during the COVID-19 pandemic, mainly on the criteria used for tiebreakers and their underlying values. The research strategy resulted in 20 publications between 2020 and 2022. A single study conducted a public consultation in 11 different countries through online questionnaires, including the following countries: United States of America (USA), Brazil, India, United Kingdom (UK), Italy, Germany, France, Australia, Spain, China, and South Korea with the participation of 5175 participants [40]. Another study included participants from both the USA and Canada [41]. Other studies conducted public consultation with a specific population: 4 studies from the USA [42–45]; 3 from the UK [46–48]; 2 from Iran [49, 50]; 2 from Australia [51, 52]; 1 study from each of the following countries: Italy [53], Switzerland [54], Netherlands [55]; Portugal [56], Thailand [57], Japan [58], and Korea [59].

Of the studies included for analysis, some consulted both the public as well as experts. When referring to experts for the purposes of this review, we consider experts to be members of health scholars or authorities, disaster specialists and/or health professionals in various medical disciplines, as well as other university or scientific professionals. Thus, we found that some studies consulted both the public and healthcare workers (15%) and others consulted both the public and experts from other specialties (15%).

Most consultations were conducted through online questionnaires (80%) and comments on a website (5%). Two studies conducted 2 sequential surveys of the same population [46, 54]. Only two studies (10%) used mixed methods for public consultation: surveys, interviews, and online deliberation [42]; interviews (experts only) and online/face-to-face deliberation [57]. And one study (5%) was based on online democratic deliberations only [47].

The results were classified according to the characteristics of the studies and according to the tiebreakers and main arguments of the public (Tables 1, 2).

**Fig. 3:**
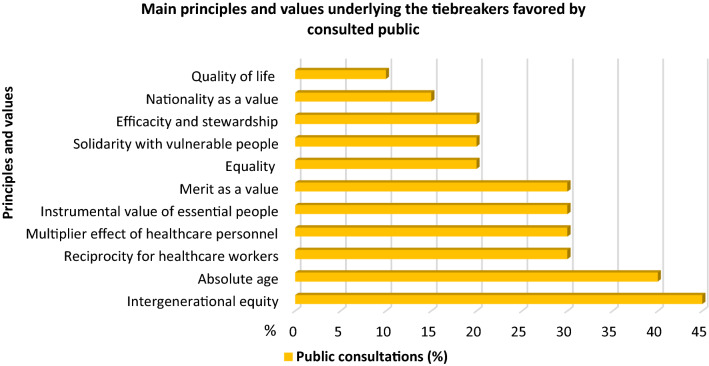
Principles and values favored by the consulted public

The principles and values considered in these public consultations are presented in percentages according to the findings found in the selected articles (Fig. 3).

Some interesting values emerged from the public consultations: consideration of the merit as a value, preference for patients affected by the pandemic disease and preference for citizens of the same nationality.

**Fig. 4:**
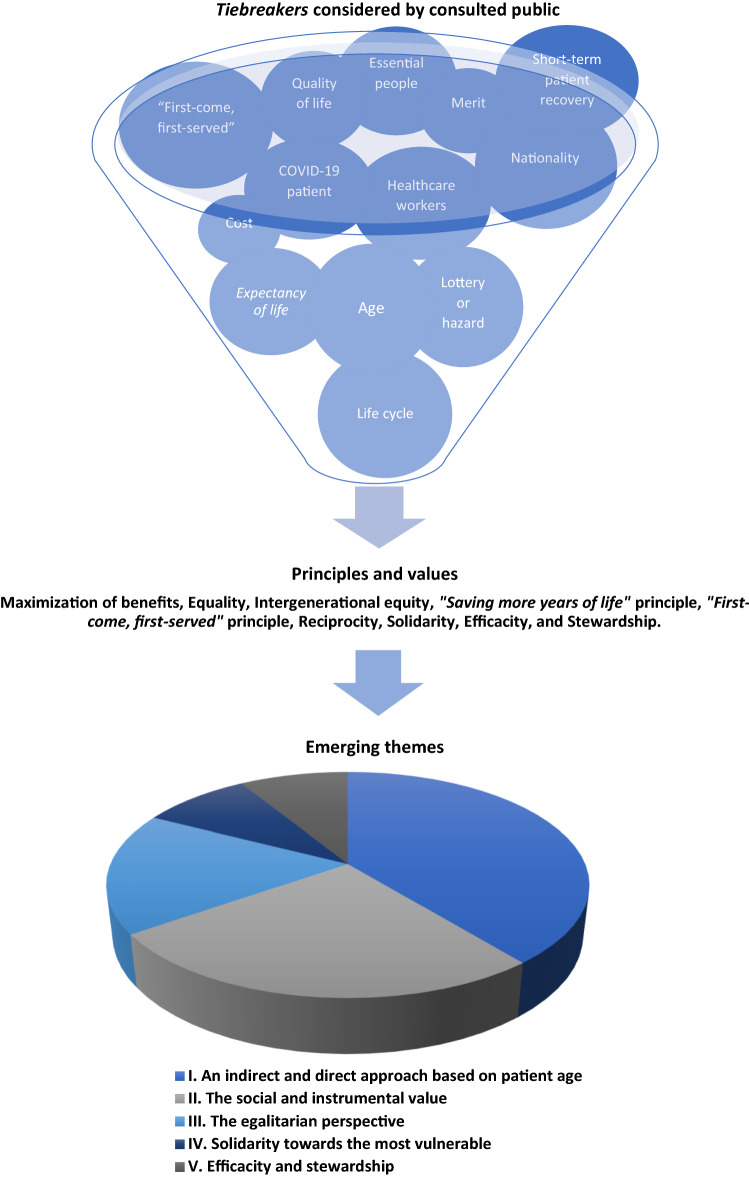
Thematic Analysis Process

From our thematic analysis, 5 themes and 15 sub-themes emerged: I. An indirect and direct approach based on patient age (intergenerational equity, absolute age and the "saving more years of life” principle); II. Social and instrumental value: essential healthcare workers (reciprocity and the multiplier effect), essential non-healthcare personnel, merit, and nationality of patient; III. The egalitarian approach (randomization and "first-come, first-served" principle); IV. Solidarity approach towards the vulnerable (pandemic disease and quality of life), and V. The efficiency and stewardship (short-term patient recovery and social/human cost) (Fig. 4).

## Discussion

This review allowed us to obtain an overview of the public's perspectives on the tiebreakers. The emerging themes and sub-themes were the product of our analysis of principles and values underlying the tiebreakers considered by the public consulted. It was interesting to note the variability in public preferences on certain tiebreakers, and the influence of culture, economic status, religious beliefs, and other values characteristic of certain populations on pandemic prioritization [40, 58, 59]. This variability of principles and values among the public has been evident in other public consultations linked to health resource allocation in pandemic, such as influenza [13, 27, 29–32]. Differences in perspectives between the public and experts on these criteria have also been found [16, 27, 28]. We present the discussion of our emerging themes and subthemes.

### An indirect and direct approach based on patient age

The patient age related to the prioritization and the tiebreakers for the allocation of scarce ICU resources during this pandemic was one of the most discussed criteria in the public consultations found in this review. To maximize the benefits in the face of a shortage of health resources, some experts have considered the patient's age to be relevant in extreme situations [60, 61]. When analyzing the results, we noted that most of the public consulted also considered age to be an important tiebreaker, both directly and indirectly. There was a slight tendency to justify it based on intergenerational equity.

#### Intergenerational equity

Intergenerational equity has been considered and discussed in several public consultations (90%) [44–47, 50–52, 55, 56, 58, 59]. This type of equity is part of the equity concepts related to health outcomes, represented by the ‘‘fair innings’’ argument when considering the criterion of the human life cycle [62]. The life cycle has been considered as a tiebreaker in triage protocols since before the appearance of COVID-19 [14, 24, 26, 63]. This criterion was also much discussed in the influenza pandemic by both the public and experts [16, 63].

According to the studies found, the public tends to favor younger patients under the perspective of life cycle, so this preference is not new. This preference is based on the premise that younger patients have yet to live their life stages, unlike older patients who have already had this opportunity, emphasizing an equality of opportunity. We believe that this criterion indirectly considers the biological age of the patient. For experts, the age considered indirectly in the life cycle is relevant to maximize the benefits in a pandemic situation, thus it was recommended to include it in the triage algorithms [9, 60, 61, 64, 65]. Because of the commitment to provide equitable access to rare resources in intensive care and to avoid discrimination based on the absolute age of the patient, the life cycle was considered a tiebreaker. Under the rationale of providing an equal opportunity to experience life, this premise could give the privilege to the youngest to be prioritized in the context of a pandemic [9, 26, 60–62, 64–66]. Of the total number of public consultations, (45%) favored the life cycle criterion [40, 44, 46, 51, 52, 55, 56, 58, 59]. In (10%) of these consultations, the participants considered it arbitrary [47, 50]. This criterion has also been considered discriminatory against the elderly [67, 68].

On the other hand, this criterion has been reformulated under the perspective of intergenerational solidarity [69]. We found only one study compatible with this perspective: the Japanese seem to favor the prioritization of the youngest by (80%) to avoid population decline (at the productive and generational level). This could be explained by the large number of senior citizens that make up its population [58]. The protection of future generations would be expressed in the value of intergenerational solidarity, by favoring the progeny considered vulnerable, and by the instinct of preservation and protection, an aspect that characterizes the human being and other living beings.

#### The stage of pregnancy

A high priority for pregnant women was also considered in two public consultations [46, 49]. However, their arguments were not elucidated. We wonder if there could be a feeling of protection towards the embryo/fetus to complete the life stages or under another perspective. This can be a controversial issue, as the legal status of the embryo/fetus can vary according to the laws of each country or state [70]. A multiplier effect perspective has been considered [70, 71]. Public consultations to try to explain the values to be considered at this stage of a woman’s reproductive cycle are necessary.

#### Absolute age

In this review, absolute age refers to considering directly and solely biological or chronological age as a tiebreaker between similar patients. It should be noted that in the literature, we found conceptual differences between biological age and chronological age that may even have legal repercussions in some countries [72, 73]. For example, biological age is programmed and modulated by internal and external factors, such as genetics and environmental exposure (lifestyle, diet, and stress among others). This is also known as physiological age, and epigenetics plays a fundamental role. While chronological age refers only to the time that elapses from the beginning of our life to the present moment, it does not necessarily correspond to biological age since biological age could be predictive of death and could be quantified by several specific biomarkers [74].

In (40%) of public consultations, the participants favored the criterion of absolute age [40, 41, 48, 49, 52, 53, 56, 59]. We believe that in some consultations, the public considered age to be important both directly and indirectly [40, 56]. In two consultations we did not identify under what values they justified giving priority to the youngest [52, 59]. In one consultation, the public indicated not to consider patient age as a prioritization criterion [54]. However, other participants considered absolute age as a tiebreaker. Their arguments focused on the fact that patient age is related to other important factors such as: prognosis, life expectancy and quality of life [46]. Others emphasized that the patient's age may influence prognosis [56]. These findings lead us to consider that the public was shocked by the high mortality rate of elderly individuals during the pandemic. Unfortunately, this vulnerable group was initially the most affected. Some believe that the effect of subjective threat may have greatly influenced the public's perspective on the relevance of certain personal characteristics of patients, including age [40]. We also share this point of view, because the uncertainty of the evolution of the pandemic and the emergence of several COVID-19 viral variants could have caused a negative impact on the public's perspectives regarding the evolution of these vulnerable groups.

These public perspectives are consistent with the scientific literature reported during the pandemic regarding elderly patients and COVID-19. In this pandemic, the most affected population initially consisted of the elderly, the immunosuppressed, and those affected by chronic comorbidities [75–77]. The mortality rate for these groups of patients was high, although the highest was in the elderly group, especially during the first two pandemic waves [78–81]. The probability of early death in intensive care was related to patient age as well, occurring more rapidly in elderly patients, because it was associated with frailty and comorbidity [78, 79, 82, 83]. In one of the public consultations, participants mentioned that elderly patients could incur premature death in the ICU, therefore age was important for them [51]. Another very important feature of the COVID-19 pandemic that influenced the high occupancy of patients in the ICU was the prolonged recovery time of patients affected by COVID-19 under the use of an invasive ventilator, which was longer than in the influenza pandemic. This prolonged time was reported to be between 2 to 4 weeks more resulting in poor accessibility for other affected patients [84, 85].

The absolute age as a tiebreaker (without specifying biological or chronological age) was rejected in (15%) of public consultations. They do not consider it fair for the elderly, but rather consider it discriminatory [47, 50, 57]. Others apparently had different perspectives when taking absolute age into account [54]. This would agree with most published protocols for accessing the ICU who do not directly take biological age or chronological age, as a single and isolated criterion in the prioritization of patients. Some take it into account indirectly as the life cycle, emphasizing intergenerational equity [86–89].

Some American protocols were modified in terms of age-linked criteria that had previously been planned in their guidelines for the allocation of scarce resources in intensive care, apparently due to criticisms [90]. Some countries in Europe also initially considered age to be important (they contemplated age limits for admission to the ICU) among their clinical prioritization criteria in their guidelines [91–93]. Their protocols were adjusted to state that age should be considered along with other important clinical criteria such as prognosis and patient comorbidity [23, 93, 94]. In Italy, the public did not agree with the pre-set age limit of a clinical assessment tool and suggested that it was less arbitrary to consider the patient's age globally [53]. It is noteworthy that some clinical tools take the patient’s age as one of their variables [25, 95]. This could be explained by the fact that the biological age of a patient influences the response to intensive therapy. The recovery time in intensive care may be longer for an elderly patient compared to a young patient [77].

On the other hand, considering the absolute age as a tiebreaker is not well seen in some countries. For example, in Japan, age is correlated to the biological sex of the patient, as the survival rate for women is higher than that of men. For this reason, the Japanese think it is a discrimination of age and biological sex at the same time. Also, the elderly have a high social and cultural value, underlining their respect and consideration. Although the public consulted prefers to give priority to the younger patient in the case of a tiebreaker, the authors believe that this guideline acceptance is difficult because of their cultural roots [58]. There is a variation in public perspectives according to their geographic location, which is generally influenced by culture and beliefs. These perspectives could disadvantage younger patients when it comes to prioritization, and this has been observed in some Eastern countries, e.g., China and Korea [40].

#### The principle of “saving more years of life”

It appears that the meaning of this principle varies among the public and has not been well defined in the literature. However, some have recently discussed and detailed their strategy for applying this principle in their prioritization protocols [65]. Others believe that there is a lack of consensus among ethicists in referring to years of life and some age-related concepts in the allocation of health resources in intensive care for this pandemic, which could lead to confusion, according to a recent review of guidelines [90]. A public consultation conducted in the context of the influenza pandemic revealed that for some public participants this principle was considered from a utilitarian perspective: to obtain more long-term benefits by seeking longer patient survival after intensive treatment [27]. Likewise, we found one study where this meant for participants to categorically exclude patients with limited remaining years of life (e.g., very old patients with very severe comorbidities) [45]. In Thailand there is a calculation for this: the “number of years of life saved” is obtained by calculating the life expectancy at birth minus the current age of the patient [57]. One study showed that the public would take this principle into account under the premise of maximizing benefits, but only as a tiebreaker [45].

### The social and instrumental value

#### Between reciprocity and the multiplier effect of essential healthcare personnel

These values towards the essential healthcare personnel were also considered in several studies (60%) [41–43, 46, 48–52, 56–58]. The public was grateful to the healthcare personnel most exposed during the pandemic. In the case of a tie, they would be willing to prioritize them for their dedication and sacrifices made for the most affected patients. The values for reciprocity and solidarity were manifested in (30%) of consultations [41, 42, 46, 48, 50, 58]. There was one study that considered prioritizing healthcare workers before considering other tiebreakers [41]. These values were compatible with experts and prioritization guidelines in which these healthcare workers are supported not only for the risk they face, but also for exposing their families to risks inherent to their work [4, 6, 9, 69, 70, 96, 97]. Priority to essential healthcare workers based on reciprocity is to acknowledge that they have suffered a disproportionately high risk during this pandemic for the good of society and are therefore deserving of priority access to scarce ICU resources in the face of a tie [7, 96, 98, 99]. However, the priority of access to critical care resources under the value of reciprocity in the face of a tie continued be debated [100, 101].

The public also considered the instrumental value of healthcare personnel in (30%) of consultations, especially those on the frontlines, as a multiplier effect of benefits. They thought about the possibility of these healthcare workers saving more human lives after recovering from their illness [42, 43, 46, 49, 50, 56]. However, this possibility does not consider the uncertainty of returning to work after hospitalization, an observation found in the literature [101, 102]. The public did not agree to prioritize other healthcare workers who have not been directly exposed to COVID-19 patients [42, 46, 50]. These findings are consistent with the results of public consultations conducted in previous pandemics in which less exposed healthcare workers would be less prioritized [27].

The public also considered the difficulty in selecting the most exposed healthcare personnel in this pandemic. Therefore, they considered this prioritization as not feasible, which could lead to a lack of trust in the management of scarce resources [57]. This is consistent with the controversy of operationalizing this criterion [103, 104]. We did not find the underlying values of this criterion in two public consultations. They also favored the most exposed healthcare personnel [51, 52]. However, in two consultations, participants considered both the value of reciprocity and the value of the multiplier effect of essential healthcare workers [42, 46].

#### Instrumental value of essential people

The value of these people was also considered by the public as a tiebreaker, in some consultations (40%) [46, 48–52, 57, 58]. For the public, it was important to give priority to people caring for children or other vulnerable people. In other words, this value was considered as an instrumental value justified by the essential character of these patients for the benefit of their dependents [46, 48, 49, 51, 52, 58]. However, other participants considered that equal opportunity should be given to people who do not have dependents [50, 57]. The Thai public argued that they did not agree with considering the social value of patients in this pandemic, as they considered it too arbitrary. Some representatives of religious groups expressed this disagreement by stating that the value of life is the same in humans [57]. Life has an equal value in every human being, however in crisis situations where available resources are limited, it is not possible to guarantee a place for all those affected. This reality cannot be ignored, so the only option is to decide who will be given priority in the face of a shortage. Here, the distributive justice plays its role in allocating these scarce resources [105]. The value of life does not change. What changes is the opportunity to save lives, and the hardest part will be to attribute this opportunity in the most objective way. This highlights the continuing controversy regarding these criteria. Perhaps the social value of each human being is one of the most debated for both the public and experts in the allocation of health resources.

On the other hand, other tiebreakers considered were prioritization based on merit and prioritization according to the patient's nationality, which were classified as part of the societal value.

#### Merit as a value

##### Individual and collective health behavior

The merit-based value of the patient was divided into two perspectives (individual and collective health behavior). The one based on individual behavior was represented by people with a history of having complied with prevention and containment measures during this pandemic and who nevertheless caught the disease. These people with COVID-19 who complied with their doctor's instructions, including compliance with the indicated treatment, and who avoided infecting others as much as possible (avoiding unnecessary high-risk behaviors, including smoking) [51, 54, 55]. The perspectives of the public consulted in terms of considering smoking as a high-risk behavior agree with the WHO, which lists smoking as a modifiable risk factor, considering it an unhealthy lifestyle, both individually and collectively, in view of the negative impacts on health (cardiovascular and oncological) [106]. These perspectives are related to studies before COVID-19 in which the public disfavored the prioritization of patients with risky lifestyle behaviors in the face of a shortage of healthcare resources [107, 108].

The second perspective was based on collective behavior, which includes people who played an important voluntary role in society during this pandemic and who became infected with the disease, such as volunteers. They would be highly prioritized by the public for their merit as well [54]. Non-healthcare personnel or individuals considered essential according to society (caregivers, policemen, soldiers, etc.) also play a great altruistic role [42, 43]. Similarly, "Good Samaritans" put their lives at risk for strangers in a selfless manner (e.g., organ donors). Experts highlight the importance of the altruism of these individuals [9, 109]. There is a merit that stands out in all these individuals, so we wonder to what extent we should ignore this merit in them, and in the case of not ignoring it, how they can be adequately rewarded?

We found no public consultation in this review that emphasized the importance of COVID-19 vaccination status for consideration as a tiebreaker. However, we did find recent publications in which this aspect was important to the public in prioritizing intensive care patients during this pandemic. This may be a subject of debate and could also be labeled as a merit of the patient in prioritizing health resources [110, 111].

##### In healthcare workers

On the other hand, we found different perspectives on the approach to the value of merit among healthcare workers that were interesting to learn about. For healthcare workers, the most exposed in this pandemic, risking their lives is part of their work (including professionals and non-professionals: doctors, nurses, firefighters, and ambulance workers, among others). This could be one of the most remarkable qualities of their vocation, which seems to be a selfless vocation. This disinterested vocation was highlighted by Japanese healthcare workers with their slogan: *Messhi houkou*, which shows that not all healthcare workers share the desire for a reward. These healthcare workers disagreed with the public regarding their prioritization in the event of a tie [58]. This was also observed in a deliberative process in North America, where it was controversial among healthcare workers themselves: while one accepted being prioritized, another indicated the opposite, although patients and caregivers were eager to prioritize them [42]. However, for some experts there is a social duty to the most exposed healthcare workers in this pandemic [99]. Regardless of whether they want to be recognized for their noble work, this social duty could be a recognition of their merit for reasons of reciprocity. This pandemic led to an unexpected overload of work for them, affecting their work performance, work/life balance, health, and quality of life.

##### In persons with a criminal record

The public gave importance to the value of merit as a tiebreaker, in such a way that the last to be prioritized as having the lowest societal merit were people with a judicial record, those involved in violent crimes and those with psychiatric problems [40, 41, 46]. Although we did not find explicit arguments about this preference, it is nothing new for these people to be rejected by society or considered undesirable groups due to their criminal records. This preference to deprioritize them was also observed in a deliberative process for the influenza pandemic [27]. We consider that this value of merit is part of the moral value of the public, who want to reward good behaviors as well as penalize misconducts at both the individual and collective level. We could say that this reward culture is learned at an early age from one’s home environment and is reinforced by society during life.

#### Nationality as a value

The nationality of the patient is a citizen characteristic whose societal value is variable between countries. There are countries with a marked nationalism and patriotism [112]. Consultations showed that the public in the case of a tie between patients, would give priority to the patient of the same nationality and not to a foreigner [40, 54, 59]. We do not know under what arguments they based their preferences. Only one study indicated impartiality to this characteristic [49]. The immigratory status of individuals should ideally not be considered when granting a live-saving health resource. Considering the patient nationality could go against the right to equality. Precisely this was one of the most discussed problems in this pandemic, trying to avoid both discrimination and social stigma among immigrants [69, 113]. The consideration of patient nationality may depend on the regulations and laws of each country; we do not know to what extent this would be allowed in certain societies. It could be an interesting subject to investigate. In America, migrant minorities (e.g., African-Hispano-Asian Americans, among others) were also the most affected in this pandemic. Several of them required intensive care with a high risk of mortality, which was related to their precarious socioeconomic situation and their underlying chronic comorbidities [114, 115]. However, the perception expressed by migrant minorities regarding unequal treatment in the prioritization of health services in non-pandemic and pandemic situations is not a new problem [116, 117].

### The egalitarian perspective

#### Between the "first-come, first-served" principle and randomization

We found controversy regarding this egalitarian perspective. The public showed weak support by considering the randomization criteria and the "first come, first served" principle in both primary and secondary prioritization as irrelevant [43, 47, 48, 53, 56, 58, 59]. Some considered that randomization is not the best way to distribute health resources, especially when lives are at stake [47]. Some expressed a preference for applying the "first come, first served" principle as a tiebreaker rather than randomization [43, 47, 59]. While for other participants, the "first come, first served" principle was considered unfair because of its association with a patient's socioeconomic situation, and would exacerbate social injustices [53, 58]. Randomization was considered the tiebreaker of last resort, after other non-clinical criteria had been weighed. For example, in a public consultation, participants suggested a coin flip as a last resort to allocate the last available resource in the ICU [48]. According to experts, randomization can be used as a tiebreaker [118, 119]. For others, randomization means absolute equality, so they would consider it in the primary prioritization [120, 121]. Randomization in practice is likely to be the least biased form of allocation and the easiest to apply, but it may also be the most uncertain in terms of its results. A comparative study based on a hypothetical simulation of pandemic influenza resulted in high patient mortality. They observed this by applying the “first-come, first-served principle”, as well as randomization for access to adult intensive care [122].

### Solidarity with vulnerable people

We found differences among the participants of the public consulted regarding the prioritization of people considered most vulnerable according to each society [40, 41, 47–49]. Two consultations showed that the public should give priority to the elderly [41, 47]. A benevolent ageism perspective could explain this priority in the face of scarce resources in pandemics by considering their vulnerability [123]. This preference can also be explained by its cultural foundations, especially in some Eastern countries [40]. Others would give priority to immunodeficient patients, although it was not specified whether it would be the primary or secondary prioritization, but a low priority to those in a terminal phase of the disease [49]. Others would also prioritize the disabled and ethnic minority groups, to not further neglect these population groups, however under certain conditions [47, 48]. For example, priority would be given to a vulnerable patient with a good quality of life [47]. In the primary prioritization, fragile patients could be considered, but in the case of a tie, they would support the less fragile patient [48]. However, frailty should not only be associated with the advanced age of the patient, because young patients under certain health conditions or comorbidities can be considered frail as well. Experts recommend that when using frailty assessments, these population groups should be well characterized [124]. On the contrary, others would give priority to patients who do not have a physical, neurodevelopmental, or mental handicap, although no arguments were found to justify this preference [51]. These findings are not new in the literature and have remained controversial [125, 126].

#### Quality of life

The public also mentioned the patient's quality of life criteria in some consultations [46, 47, 50]. This can be a controversial criterion to be considered as a tiebreaker due to its subjective character not precisely associated with the patient's age as expressed by the public [46, 47]. Health-related Quality of Life (HRQOL) and Quality Adjusted Life Years (QALYs) assessments have been considered unsuitable to include in the prioritization criteria because of the risk of increasing discrimination between the disabled and the elderly [16, 64]. For some experts, people perceive their own quality of life differently from the perceptions of others. There have been discrepancies in the perception of quality of life between the evaluators and the self-evaluations of people with disabilities [127, 128]. The public was hesitant to consider this criterion because it is associated with the socioeconomic situation of the patient as well, in order not to accentuate social inequalities. So, they suggested considering it as a tiebreaker as a last resort [47]. In Iran, the public disagreed in evaluating the patient's quality of life for prioritization, so the consideration as a tiebreaker was not widely supported [50].

#### Pandemic disease priority

Giving priority to the patient affected by a pandemic disease such as COVID-19 over another type of disease was a preference expressed by the public in two studies [41, 43]. We do not know the reasons for this, but it may be related to the uncertain evolution of this pandemic, i.e., the severity of the pandemic situation may have influenced the emergence of these perspectives, either to control it or to pay more attention to vulnerable victims. The rest of the public consultations found do not make mention of this distinction. It is important to note that most guidelines for accessing the ICU in this pandemic agree not to distinguish between a COVID-19 and a non-COVID-19 patient, because in both cases life is at stake. The patient's prognosis depends not only on the acute disease (pandemic disease). It also depends on other acute, chronic, and mixed clinical criteria [23, 69, 119].

### Efficacity and stewardship

#### Short-term patient recovery

This criterion was also mentioned by the public as important to consider when faced with the impasse of deciding between two patients with similar survival prognosis. They prefer to give the opportunity to the patient who is likely to last less time in intensive care, to optimize the use of the few resources available and to speed up access to other patients in need [47, 48, 58]. This criterion is very relevant and at the same time debatable, when considering that this short-term recovery may be associated with other clinical characteristics of the patient, such as biological age, the presence of comorbidities, innate components (genetic/hereditary), among others. The value of this criterion could be interpreted as efficiency in the administration of scarce healthcare resources, a procedural value also contemplated in most guidelines and with a utilitarian orientation [17, 23, 53].

#### Financial and human cost

The public also expressed a preference for reducing social costs. This cost was considered from two perspectives by the public: a financial perspective based on the reduction of economic costs combined with efficiency in resource management. They considered it important to reduce the costs incurred by patients admitted to the ICU in the face of a pandemic shortage. They suggested prioritizing patients who spend less time on an invasive ventilator. The other perspective was related to the irresponsible behavior of the patient in contaminating other people, increasing the risk of contagion and of human loss (a human cost) [40].

These values considered by the public lead us to think that they have also reasoned on how to manage scarce health resources to preserve more human lives, a tendency to maximize benefits in an extreme context. We could consider in a certain way a transition from the individual to the collective perspective. This would be consistent with most of the protocols for access to the ICU in a pandemic situation [17, 23, 86].

### Looking for a consensus

According to our results, it is not easy to obtain a consensus, especially when there is a heterogeneity in public perspectives [57]. Some consider that homogeneity of perspectives in a community is difficult to find due to multiple social, cultural, and economic differences internally. [32]. One of the few studies that indicated a consensus on tiebreakers was the prioritization of the most exposed healthcare personnel by consensus of patients and caregivers, although this consensus was not achieved with the experts in the deliberations [42]. In Japan and Thailand, it was unrealistic for the public to obtain a consensus [57, 58]. In some consultations, there was no concordance between the perspectives expressed by the public on prioritization and their triage guidelines [46, 48, 58]. It seems that a few studies did observe this concordance of perspectives with their triage protocols [51, 52].

Further studies are needed to explain the discrepancies between the public and experts regarding prioritization in this pandemic. Consultations conducted through deliberative processes during this pandemic are very rare. We consider that this would be one of the most outstanding gaps: the search for an optimization of COVID-19 protocols thanks to citizen deliberation. Another aspect is the absence of public consultations of certain groups such as indigenous populations, and of other nationalities, notably from countries with low or medium incomes where these countries are the most disadvantaged in a pandemic. There is little information on the prioritization of pregnant women in pandemic contexts.

## Limitations

The research strategy was limited to 3 languages (English, French and Spanish). Despite having reformulated our research strategy to obtain more articles of interest in the databases, we observed that there is very little literature on the subject. Our search in the gray literature was not exhaustive considering this. We do not rule out the possibility that several studies have not yet been published during our search. A methodological assessment of bias of the selected articles was not a methodological requirement in this review, so it was not performed [36, 38]. Our objective was mainly to find public arguments regarding their preference for tiebreakers and values. Only 3 consultations carried out deliberative processes, which quoted the arguments of the public and allowed the capture of their expressions [42, 47, 57]. This somewhat limited our qualitative analysis of the perspectives of the public consulted. Most of these perspectives were obtained from the results described by the authors of articles. We are aware that the public perspectives found in this review may be modulated during this pandemic, so this interpretation cannot be generalized.

## Conclusion

This scoping review provides an overview of what the public indicated was important to consider as a tiebreaker in the decision to admit patients to intensive care in the context of this pandemic. We focused our analysis according to the principles and values underlying these criteria mentioned by the public consulted. We obtained five emerging themes from our thematic analysis. This scoping review shows that patient age was the most discussed criterion, and that there is a preference for favoring younger patients over older patients when there is a tie between patients with similar prognoses, with a slight tendency to favor intergenerational equity. The social and instrumental value of individuals was also another of the themes that stood out. Among the interesting tiebreakers considered by the public were the recognition of the merit of the patient, the preference for nationality, and for those affected by the pandemic disease, although their arguments were not well defined. Variability was found in public perspectives in relation to the tiebreakers and their values. This variability was related to socio-cultural, and religious factors. We observed that tiebreakers continue to be a source of debate for both the public and experts, as some consultations were made in both groups. More studies are needed to understand the public's perspective on tiebreakers. We hope that these collected public perspectives can be useful in the development or optimization of triage criteria for admission to adult intensive care in the context of a pandemic.

## Supplementary Information

Below is the link to the electronic supplementary material.

Supplementary file1 (DOCX 690 KB)

Supplementary file2 (DOCX 18 KB)

## Data Availability

All the data and materials of this study (Tables, Figures, Geographic Map, and Additional Files) are incorporated within this publication.

## Abbreviations

COVID-19: Coronavirus disease 2019
ICU: Intensive care unit
WHO: World Health Organisation
HRQOL: Health-related quality of life
QALYs: Quality adjusted life years
USA: United States of America
UK: United Kingdom

## References

[1] WHO Director-General’s opening remarks at the media briefing on COVID-19. 2020. https://www.who.int/dg/speec hes/detail/who-director-general-s-opening-remarks-at-the-media-brief ing-on-covid-19–11-march-2020.

[2] Downar J, Smith MJ, Godkin D, Frolic A, Bean S, Bensimon C, Bernard C, Huska M, Kekewich M, Ondrusek N, Upshur R, Zlotnik-Shaul R, Gibson J. A framework for critical care triage during a major surge in critical illness. Can J Anaesth. 2022;69(6):774–81. 10.1007/s12630-022-02231-2. 35322377 10.1007/s12630-022-02231-2 PMC8942150

[3] Bauer J, Brüggmann D, Klingelhöfer D, Maier W, Schwettmann L, Weiss DJ, Groneberg DA. Access to intensive care in 14 European countries: a spatial analysis of intensive care need and capacity in the light of COVID-19. Intensive Care Med. 2020;46(11):2026–34. 10.1007/s00134-020-06229-6. 32886208 10.1007/s00134-020-06229-6 PMC7472675

[4] Maves RC, Downar J, Dichter JR, Hick JL, Devereaux A, Geiling JA, Kissoon N, Hupert N, Niven AS, King MA, Rubinson LL, Hanfling D, Hodge JG Jr, Marshall MF, Fischkoff K, Evans LE, Tonelli MR, Wax RS, Seda G, Parrish JS, Truog RD, Sprung CL, Christian MD. ACCP task force for mass critical care triage of scarce critical care resources in COVID-19 an implementation guide for regional allocation: an expert panel report of the task force for mass critical care and the american college of chest physicians. Chest. 2020;158(1):212–25. 10.1016/j.chest.2020.03.063. 32289312 10.1016/j.chest.2020.03.063 PMC7151463

[5] Aziz S, Arabi YM, Alhazzani W, Evans L, Citerio G, Fischkoff K, Salluh J, Meyfroidt G, Alshamsi F, Oczkowski S, et al. Managing ICU surge during the COVID-19 crisis: rapid guidelines. Intensive Care Med. 2020. 10.1007/s00134-020-06092-5. 32514598 10.1007/s00134-020-06092-5 PMC7276667

[6] White DB, Lo B. A framework for rationing ventilators and critical care beds during the COVID-19 pandemic. JAMA. 2020. 10.1001/jama.2020.5046. 32219367 10.1001/jama.2020.5046

[7] Leclerc T, Donat N, Donat A, Pasquier P, Libert N, Schaeffer E, et al. Prioritisation of ICU treatments for critically ill patients in a COVID-19 pandemic with scarce resources. Anaesth Crit Care Pain Med. 2020;39:333–9. 32426441 10.1016/j.accpm.2020.05.008 PMC7230138

[8] Vincent J-L, Creteur J. Ethical aspects of the COVID-19 crisis: how to deal with an overwhelming shortage of acute beds. Eur Heart J Acute Cardiovasc Care. 2020;9(3):248–52. 32347745 10.1177/2048872620922788 PMC7196891

[9] Emanuel EJ, Persad G, Upshur R, Thome B, Parker M, Glickman A, et al. Fair allocation of scarce medical resources in the time of Covid-19. N Engl J Med. 2020. 10.1056/NEJMsb2005114. 32202722 10.1056/NEJMsb2005114

[10] Michailidou G. Biases in COVID-19 Medical Resource Dilemmas. Front Psychol. 2021;12(12):687069. 10.3389/fpsyg.2021.687069. 34475836 10.3389/fpsyg.2021.687069 PMC8406935

[11] Kooli C. COVID-19: Public health issues and ethical dilemmas. Ethics Med Public Health. 2021. 10.1016/j.jemep.2021.100635. 33553555 10.1016/j.jemep.2021.100635 PMC7847409

[12] Orfali K. Getting to the truth: ethics, trust, and triage in the united states versus europe during the covid-19 pandemic. Hastings Cent Rep. 2021;51(1):16–22. 10.1002/hast.1206. 33486774 10.1002/hast.1206 PMC8014048

[13] Whitty JA, Lancsar E, Rixon K, Golenko X, Ratcliffe J. A systematic review of stated preference studies reporting public preferences for healthcare priority setting. Patient. 2014;7(4):365–86. 10.1007/s40271-014-0063-2. 24872225 10.1007/s40271-014-0063-2

[14] Gu Y, Lancsar E, Ghijben P, Butler JR, Donaldson C. Attributes and weights in health care priority setting: a systematic review of what counts and to what extent. Soc Sci Med. 2015;146:41–52. 10.1016/j.socscimed.2015.10.005. 26498059 10.1016/j.socscimed.2015.10.005

[15] Leider JP, DeBruin D, Reynolds N, Koch A, Seaberg J. Ethical guidance for disaster response, specifically around crisis standards of care: a systematic review. Am J Public Health. 2017;107(9):e1–9. 10.2105/AJPH.2017.303882. 28727521 10.2105/AJPH.2017.303882 PMC5551597

[16] Ghanbari V, Ardalan A, Zareiyan A, Nejati A, Hanfling D, Bagheri A, Rostamnia L. Fair prioritization of casualties in disaster triage: a qualitative study. BMC Emerg Med. 2021;21(1):119. 10.1186/s12873-021-00515-2. 34645418 10.1186/s12873-021-00515-2 PMC8513386

[17] Fiest KM, Krewulak KD, Plotnikoff KM, et al. Allocation of intensive care resources during an infectious disease outbreak: a rapid review to inform practice. BMC Med. 2020;18:404. 10.1186/s12916-020-01871-9. 33334347 10.1186/s12916-020-01871-9 PMC7746486

[18] Iacorossi L, Fauci AJ, Napoletano A, D’Angelo D, Salomone K, Latina R, Coclite D, Iannone P. Triage protocol for allocation of critical health resources during Covid-19 pandemic and public health emergencies. A Narrative Review Acta Biomed. 2020;91(4):e2020162. 10.2750/abm.v91i4.10393. 33525236 10.23750/abm.v91i4.10393 PMC7927504

[19] Piscitello GM, Kapania EM, Miller WD, Rojas JC, Siegler M, Parker WF. Variation in ventilator allocation guidelines by us state during the coronavirus disease 2019 pandemic: a systematic review. JAMA Netw Open. 2020;3(6):e2012606. 10.1001/jamanetworkopen.2020.12606. 32558916 10.1001/jamanetworkopen.2020.12606 PMC7305526

[20] Tyrrell CSB, Mytton OT, Gentry SV, Thomas-Meyer M, Allen JLY, Narula AA, McGrath B, Lupton M, Broadbent J, Ahmed A, Mavrodaris A, Abdul Pari AA. Managing intensive care admissions when there are not enough beds during the COVID-19 pandemic: a systematic review. Thorax. 2021;76(3):302–12. 10.1136/thoraxjnl-2020-215518. 33334908 10.1136/thoraxjnl-2020-215518 PMC7892390

[21] Cleveland Manchanda EC, Sanky C, Appel JM. Crisis Standards of Care in the USA: A Systematic Review and Implications for Equity Amidst COVID-19. J Racial Ethn Health Disparities. 2021;8(4):824–36. 10.1007/s40615-020-00840-5. 32789816 10.1007/s40615-020-00840-5 PMC7425256

[22] Perin M, De Panfilis L. Among equity and dignity: an argument-based review of European ethical guidelines under COVID-19. BMC Med Ethics. 2021;22(1):36. 10.1186/s12910-021-00603-9. 33789633 10.1186/s12910-021-00603-9 PMC8011067

[23] Aquino YSJ, Rogers WA, Scully JL, Magrabi F, Carter SM. Ethical guidance for hard decisions: a critical review of early international covid-19 icu triage guidelines. Health Care Anal. 2022;30(2):163–95. 10.1007/s10728-021-00442-0. 34704198 10.1007/s10728-021-00442-0 PMC8547561

[24] dos Santos MJ, Martins MS, Santana FLP, et al. COVID-19: instruments for the allocation of mechanical ventilators—a narrative review. Crit Care. 2020;24:582. 10.1186/s13054-020-03298-3. 32993736 10.1186/s13054-020-03298-3 PMC7522926

[25] Cardona M, Dobler CC, Koreshe E, Heyland DK, Nguyen RH, Sim JPY, Clark J, Psirides A. A catalogue of tools and variables from crisis and routine care to support decision-making about allocation of intensive care beds and ventilator treatment during pandemics: Scoping review. J Crit Care. 2021;66:33–43. 10.1016/j.jcrc.2021.08.001. 34438132 10.1016/j.jcrc.2021.08.001

[26] Frolic A, et al. Development of a Critical care triage protocol for pandemic influenza: integrating ethics. Evidence and Effectiveness: Healthcare Quarterly; 2009. 10.1927/hcq.2009.21054. 10.12927/hcq.2009.21054 20057230

[27] Biddison ELD, Gwon HS, Schoch-Spana M, Regenberg AC, Juliano C, Faden RR, et al. Scarce resource allocation during disasters: a mixed-method community engagement study. Chest. 2018;153(1):187–95. 28802695 10.1016/j.chest.2017.08.001

[28] Krütli P, Rosemann T, Törnblom KY, Smieszek T. How to fairly allocate scarce medical resources: ethical argumentation under scrutiny by health professionals and lay people. PLoS ONE. 2016;11(7): e0159086. 27462880 10.1371/journal.pone.0159086 PMC4963105

[29] Silva DS, Gibson JL, Robertson A, et al. Priority setting of ICU resources in an influenza pandemic: a qualitative study of the Canadian public’s perspectives. BMC Public Health. 2012;12(1):241. 22449119 10.1186/1471-2458-12-241 PMC3331804

[30] Smith M, Bensimon C, Perez D, Sahni S, Upshur R. Restrictive measures in an influenza pandemic: a qualitative study of public perspectives. Can J Public Health. 2012;103(5):e348–52. 10.1007/bf03404439. 23617986 10.1007/BF03404439 PMC6973992

[31] Charania NA, Tsuji LJS. A community-based participatory approach and engagement process creates culturally appropriate and community informed pandemic plans after the 2009 H1N1 influenza pandemic: remote and isolated first nations communities of subarctic Ontario. Canada BMC Public Health. 2012;12:268. 22472012 10.1186/1471-2458-12-268 PMC3434059

[32] Chiam AL, Cheng NWI, Larson H. Community engagement for outbreak preparedness and response in high-income settings: a systematic review. Glob Public Health. 2021;2:1–23. 10.1080/17441692.2021.1919734. 10.1080/17441692.2021.1919734 33938368

[33] Farmer Y, Bouthillier MÈ, Dion-Labrie M, Durand C, Doucet H. Public participation in national preparedness and response plans for pandemic influenza: Towards an ethical contribution to public health policies. Ramon Llull J Appl Ethics. 2010;1:9–23.

[34] Kim S, Wall I, Stanczyk A, De Vries R. Assessing the public’s views in research ethics controversies: deliberative democracy and bioethics as natural allies. J Empir Res Hum Res Ethics. 2009;4(4):3–16. 10.1525/jer.2009.4.4.3. 19919315 10.1525/jer.2009.4.4.3 PMC2853797

[35] Solomon S, Abelson J. Why and when should we use public deliberation? Hastings Cent Rep. 2012;42:17–20. 22733325 10.1002/hast.27 PMC3645483

[36] Arksey H, O’Malley L. Scoping studies: towards a methodological framework. Int J Soc Res Methodol. 2005;8:19–32.

[37] Levac D, Colquhoun H, O’Brien KK. Scoping studies: advancing the methodology. Implement Sci. 2010;5:69. 20854677 10.1186/1748-5908-5-69 PMC2954944

[38] Parsons JA, Johal HK. In defence of the bioethics scoping review: largely systematic literature reviewing with broad utility. Bioethics. 2021. 10.1111/bioe.12991. 34969147 10.1111/bioe.12991

[39] Tricco AC, Lillie E, Zarin W, et al. PRISMA extension for scoping reviews (PRISMA-ScR): checklist and explanation. Ann Intern Med. 2018;169:467–73. 30178033 10.7326/M18-0850

[40] Jin L, Huang Y, Liang Y, Zhang Q. Who gets the ventilator? Moral decision making regarding medical resource allocation in a pandemic. J Assoc Consum Res. 2021;6(1):159–67.

[41] Géa LP, Upfold C, Qureshi A, Moulden HM, Mamak M, McDonald Wilson Bradford J, Chaimowitz GA. Public perceptions of psychiatric, justice-involved, and elderly populations during the COVID-19 pandemic. J Psychiatr Res. 2022;146:67–76. 10.1016/j.jpsychires.2021.12.019. 34954362 10.1016/j.jpsychires.2021.12.019 PMC8689415

[42] Bruno B, Hurwitz HM, Mercer M, Mabel H, Sankary L, Morley G, Ford PJ, Horsburgh CC, Rose SL. Incorporating Stakeholder perspectives on scarce resource allocation: lessons learned from policymaking in a time of crisis. Camb Q Healthc Ethics. 2021;30(2):390–402. 10.1017/S0963180120000924. 33764294 10.1017/S0963180120000924

[43] Fallucchi F, Faravelli M, Quercia S. Fair allocation of scarce medical resources in the time of COVID-19: what do people think? J Med Ethics. 2021;47(1):3–6. 33046588 10.1136/medethics-2020-106524 PMC7551740

[44] Huseynov S, Palma MA, Nayga RM Jr. General public preferences for allocating scarce medical resources during COVID-19. Front Public Health. 2020. 10.3389/fpubh.2020.587423. 33363084 10.3389/fpubh.2020.587423 PMC7759519

[45] Rai B, Wang LC, Pandit S, Handfield T, So CK. Awareness of ethical dilemmas enhances public support for the principle of saving more lives in the United States: A survey experiment based on ethical allocation of scarce ventilators. Soc Sci Med. 2021. 10.1016/j.socscimed.2021.114171. 34175572 10.1016/j.socscimed.2021.114171

[46] Chan, L., et al. “Which Features of Patients Are Morally Relevant in Ventilator Triage? A survey of the UK Public. BMC Medical Ethics BioMed Central. 2022. 10.1186/s12910-022-00773-0 PMC8956145 35337310

[47] Kuylen MNI, Kim SY, Ruck Keene A, Owen GS. Should age matter in COVID-19 triage? A deliberative study 2021 Mar 9. J Med Ethics. 2021;47(5):291–5. 10.1136/medethics-2020-107071. 33687917 10.1136/medethics-2020-107071 PMC7944418

[48] Wilkinson D, Zohny H, Kappes A, Sinnott-Armstrong W, Savulescu J. Which factors should be included in triage? An online survey of the attitudes of the UK general public to pandemic triage dilemmas. BMJ Open. 2020;10(12):e045593. 10.1136/bmjopen-2020-045593. 33293401 10.1136/bmjopen-2020-045593 PMC7725087

[49] Abbasi-Kangevari M, Arshi S, Hassanian-Moghaddam H, Kolahi AA. Public opinion on priorities toward fair allocation of ventilators during COVID-19 pandemic: a nationwide survey. Front Public Health. 2021. 10.3389/fpubh.2021.753048. 34970524 10.3389/fpubh.2021.753048 PMC8712311

[50] Asghari F, Parsapour A, Shamsi GE. Priority setting of ventilators in the COVID-19 pandemic from the public’s perspective. AJOB Empir Bioeth. 2021;12(3):155–63. 10.1080/23294515.2021.1907474. 33881385 10.1080/23294515.2021.1907474

[51] Norman R, Robinson S, Dickinson H, et al. Public preferences for allocating ventilators in an intensive care unit: a discrete choice experiment. Patient. 2021;14:319–30. 10.1007/s40271-021-00498-z. 33660163 10.1007/s40271-021-00498-z PMC7929628

[52] Street AE, Street DJ, Flynn GM. Who gets the last bed? A discrete-choice experiment examining general population preferences for intensive care bed prioritization in a pandemic. Med Decis Making. 2021;41(4):408–18. 10.1177/0272989X21996615. 33660540 10.1177/0272989X21996615 PMC7941140

[53] Riccioni L, Ingravallo F, Grasselli G, Mazzon D, Cingolani E, Forti G, Zagrebelsky V, Zoja R, Petrini F. The Italian document: decisions for intensive care when there is an imbalance between care needs and resources during the COVID-19 pandemic. Ann Intensive Care. 2021;11(1):100. 10.1186/s13613-021-00888-4. 34189634 10.1186/s13613-021-00888-4 PMC8241202

[54] Knotz CM, Gandenberger MK, Fossati F, Bonoli G. Public attitudes toward pandemic triage: evidence from conjoint survey experiments in Switzerland. Soc Sci Med. 2021;285:114238. 10.1016/j.socscimed.2021.114238. 34364159 10.1016/j.socscimed.2021.114238 PMC8417366

[55] Gijsbers M, Keizer IE, Schouten SE, Trompert JL, Groothuis-Oudshoorn CGM, van Til JA. Public preferences in priority setting when admitting patients to the ICU during the COVID-19 crisis: a pilot study. Patient. 2021;14(3):331–8. 10.1007/s40271-021-00504-4. 33748930 10.1007/s40271-021-00504-4 PMC7982314

[56] Pinho M. Public preferences for allocating absolute scarce critical healthcare resources during the COVID-19 pandemic. J Health Organ Manag. 2021. 10.1108/JHOM-12-2020-0494. 34018706 10.1108/JHOM-12-2020-0494

[57] Marshall AI, Archer R, Witthayapipopsakul W, et al. Developing a Thai national critical care allocation guideline during the COVID-19 pandemic: a rapid review and stakeholder consultation. Health Res Policy Syst. 2021;19(1):47. 10.1186/s12961-021-00696-z. 33789671 10.1186/s12961-021-00696-z PMC8011047

[58] Norisue Y, Deshpande GA, Kamada M, Nabeshima T, Tokuda Y, Goto T, Ishizuka N, Hara Y, Nakata R, Makino J, Matsumura M, Fujitani S, Hiraoka E. Allocation of mechanical ventilators during a pandemic: a mixed-methods study of perceptions among japanese health care workers and the general public. Chest. 2021;159(6):2494–502. 10.1016/j.chest.2021.01.015. 33444616 10.1016/j.chest.2021.01.015

[59] Lee JS, Kim S, Do YK. Public preferences for allocation principles for scarce medical resources in the COVID-19 pandemic in Korea: comparisons with ethicists’ recommendations. J Prev Med Publ Health. 2021;54(5):360–9. 10.3961/jpmph.21.333. 10.3961/jpmph.21.333 PMC8517370 34649398

[60] Altman MC. A consequentialist argument for considering age in triage decisions during the coronavirus pandemic. Bioethics. 2021;35(4):356–65. 10.1111/bioe.12864. 33683705 10.1111/bioe.12864 PMC8251012

[61] Vinay R, Baumann H, Biller-Andorno N. Ethics of ICU triage during COVID-19. Br Med Bull. 2021;138(1):5–15. 10.1093/bmb/ldab009. 34057458 10.1093/bmb/ldab009 PMC8195142

[62] Williams A. Intergenerational equity. Health Econ. 1997;6(2):117–32. 9158965 10.1002/(sici)1099-1050(199703)6:2<117::aid-hec256>3.0.co;2-b

[63] Biddison E, Gwon FR, Mareiniss H, Regenberg D, Schoch-Spana A, Schwartz M, Toner JE. Too many patients a framework to guide statewide allocation of scarce mechanical ventilation during disasters contemporary reviews in critical care medicine. CHEST. 2019;155(4):848–54. 30316913 10.1016/j.chest.2018.09.025

[64] White DB, Katz MH, Luce JM, Lo B. Who should receive life support during a public health emergency? Using ethical principles to improve allocation decisions. Ann Intern Med. 2009;150(2):132–8. 19153413 10.7326/0003-4819-150-2-200901200-00011 PMC2629638

[65] Bouthillier M-E, Kramer A, Moreau M. Le cycle de vie pour prioriser les patients aux soins intensifs en contexte extrême de pandémie : défis éthiques et pratiques. Éthique publique. 2022. 10.4000/ethiquepublique.6675.

[66] Persad G, Joffe S. Allocating scarce life-saving resources: the proper role of age. J Med Ethics. 2021. 10.1136/medethics-2020-106792. 33753473 10.1136/medethics-2020-106792

[67] Jecker NS. Too old to save? COVID-19 and age-based allocation of lifesaving medical care. Bioethics. 2022. 10.1111/bioe.13041. 35533287 10.1111/bioe.13041 PMC9347438

[68] Brown TR, Francis LP, Tabery J. When is age choosing ageist discrimination? Hastings Cent Rep. 2021;51(1):13–5. 10.1002/hast.1205. 33320366 10.1002/hast.1205

[69] Verweij M, van de Vathorst S, Schermer M, Willems D, de Vries M. Ethical advice for an intensive care triage protocol in the COVID-19 pandemic: lessons learned from The Netherlands. Public Health Ethics. 2020;13(2):157–65. 10.1093/phe/phaa027. 33288984 10.1093/phe/phaa027 PMC7499738

[70] Critical Care Bioethics Working Group. Allocation of Scarce Critical Resources under Crisis Standards of Care. University of California. Revised June 17. 2020.

[71] Rajczi A, Daar J, Kheriaty A, Dastur C. The university of California crisis standards of care: public reasoning for socially responsible medicine. Hastings Cent Rep. 2021;51(5):30–41. 10.1002/hast.1284. 34529849 10.1002/hast.1284

[72] Lippert-Rasmussen Kasper, Petersen TS. Age change, official age and fairness in health. J Med Ethics. 2020;46:636–7. 10.1136/medethics-2020-106078. 32054777 10.1136/medethics-2020-106078

[73] Räsänen J. Moral case for legal age change. J Med Ethics. 2019;45(7):461–4. 10.1136/medethics-2018-105294. 30872325 10.1136/medethics-2018-105294

[74] Diebel LWM, Rockwood K. Determination of biological age: geriatric assessment vs biological biomarkers. Curr Oncol Rep. 2021;23(9):104. 10.1007/s11912-021-01097-9. 34269912 10.1007/s11912-021-01097-9 PMC8284182

[75] Wu C, Chen X, Cai Y, et al. Risk factors associated with acute respiratory distress syndrome and death in patients with coronavirus disease 2019 pneumonia in Wuhan. China JAMA Intern Med. 2020;30:994–7. 10.1001/jamainternmed.2020.0994 PMC7070509 32167524

[76] Wu Z, McGoogan JM. Characteristics and important lessons from the coronavirus disease 2019 (COVID-19) outbreak in china: summary of a report of 72314 cases from the Chinese center for disease control and prevention. JAMA. 2020;13:1239–42. 10.1001/jama.2020.2648 32091533

[77] Zhou F, Yu T, Du R, et al. Clinical course and risk factors for mortality of adult inpatients with COVID-19 in Wuhan, China: a retrospective cohort study. Lancet. 2020;395:1054–62. 32171076 10.1016/S0140-6736(20)30566-3 PMC7270627

[78] Iaccarino G, Grassi G, Borghi C, Ferri C, Salvetti M, Volpe M. SARS-RAS investigators age and multimorbidity predict death among COVID-19 patients results of the SARS-RAS study of the italian society of hypertension. Hypertension. 2020. 10.1161/HYPERTENSIONAHA.120.15324. 32564693 10.1161/HYPERTENSIONAHA.120.15324

[79] Institut national d’excellence en santé et en services sociaux (INESSS). Première vague de la pandémie de COVID-19 au Québec : regard sur les facteurs associés aux hospitalisations et aux décès. État des pratiques rédigé par Éric Tremblay et Mike Benigeri. Québec, Qc INESSS. 2020. 60

[80] ICNARC COVID-19 Team. Richards-Belle A, Orzechowska I, Gould DW, et al. 2020 COVID-19 in critical care epidemiology of the first epidemic wave across England Wales and Northern Ireland. Intensive Care Med. 10.1007/s00134-020-06267-0 10.1007/s00134-020-06267-0 PMC7545019 33034689

[81] Yang X, Yu Y, Xu J, Shu H, Xia J, Liu H, et al. Clinical course and outcomes of critically ill patients with SARS-CoV-2 pneumonia in Wuhan, China: a single centered, retrospective, observational study. Lancet Respir Med. 2020;8(5):475. 32105632 10.1016/S2213-2600(20)30079-5 PMC7102538

[82] Hägg S, Jylhävä J, Wang Y, et al. Age, frailty, and comorbidity as prognostic factors for short-term outcomes in patients with coronavirus disease 2019 in geriatric care. J Am Med Dir Assoc. 2020;21(11):1555-1559.e2. 10.1016/j.jamda.2020.08.014. 32978065 10.1016/j.jamda.2020.08.014 PMC7427570

[83] Price A, Barlow-Pay F, Dufy S, et al. Study protocol for the COPE study: COVID-19 in Older PEople: the infuence of frailty and multimorbidity on survival a multicentre, european observational study COPE study collaborators. BMJ Open. 2020. 10.1136/bmjopen-2020-040569. 32994260 10.1136/bmjopen-2020-040569 PMC7526029

[84] Holanda MA, Pinheiro BV. COVID-19 pandemic and mechanical ventilation: facing the present, designing the future. J Bras Pneumol. 2020. 10.36416/1806-3756/e20200282. 32696835 10.36416/1806-3756/e20200282 PMC7567632

[85] Faury H, Courboulès C, Payen M, Jary A, Hausfater P, Luyt C, Dres M, Pourcher V, Abdi B, Wirden M, Calvez V, Marcelin AG, Boutolleau D, Burrel S. Medical features of COVID-19 and influenza infection: a comparative study in Paris. France J Infect. 2021;82(2):e36–9. 10.1016/j.jinf.2020.08.017. 32798533 10.1016/j.jinf.2020.08.017 PMC7426213

[86] Joebges S, Biller-Andorno N. Ethics guidelines on COVID-19 triage-an emerging international consensus. Crit Care Lond Engl. 2020;24(1):201. 10.1186/s13054-020-02927-1 PMC7202791 32375855

[87] University of Pittsburgh Department of Critical Care Medicine. Allocation of Scarce Critical Care Resources During a Public Health Emergency. March 23. 2020. https://ccm.pitt.edu/sites/default/files/.

[88] Opartny L. et al. PRIORISATION POUR L’ACCES AUX SOINS INTENSIFS (ADULTES) EN CONTEXTE EXTRÊME DE PANDÉMIE. Version 2020–11–02. Santé et Services Sociaux du Québec.

[89] Warrillow S, Austin D, Cheung W, Close E, Holley A, Horgan B, Jansen M, Joynt G, Lister P, Moodie S, Nichol A, Nicholls M, Peake S, Skowronski G, Streat S, White B, Willmott L. ANZICS guiding principles for complex decision making during the COVID-19 pandemic. Crit Care Resusc. 2020;22(2):98–102. 32294810 10.51893/2020.2.sa1 PMC10692461

[90] Scire E, Jeong KY, Gaurke M, Prusak B, Sulmasy DP. Rationing with respect to age during a pandemic: a comparative analysis of state pandemic preparedness plans. Chest. 2022;161(2):504–13. 10.1016/j.chest.2021.08.070. 34506791 10.1016/j.chest.2021.08.070 PMC8423769

[91] SEMICYUC Sociedad Española de Medicina Intensiva, Crítica y Unidades Coronarias. Recomendaciones éticas para la toma de decisiones en la situación excepcional de crisis por pandemia COVID-19 en las unidades de cuidados intensivos (2020). https://semicyuc.org/wpcontent/uploads/2020/03/Ética.SEMICYUC-COVID-19.pdf.10.1016/j.medin.2020.04.006PMC715879032402532

[92] Vergano M, Bertolini G, Giannini A, Gristina G, Livigni S, Mistraletti G, et al. Clinical ethics recommendation for the allocation of intensive care treatments, in exceptional resource-limited circumstances. http://www.siaarti.it: SIAARTI. 2020. http://www.siaarti.it/SiteAssets/News/COVID19-documentSIAARTI/SIAARTI-Covid-19-ClinicalEthicsReccomendations.pdf. 10.23736/S0375-9393.20.14619-4 32242647

[93] Rosenbaum L. Facing Covid-19 in Italy. N Engl J Med. 2020;382:1873–5. 32187459 10.1056/NEJMp2005492

[94] Herreros B, Gella P, Real de Asua D. Triage during the COVID-19 epidemic in Spain better and worse ethical arguments. J Med Ethics. 2020. 10.1136/medethics-2020-106352. 32424063 10.1136/medethics-2020-106352

[95] Kaymak C, Sencan I, Izdes S, Sari A, Yagmurdur H, Karadas D, Oztuna D. Mortality of adult intensive care units in Turkey using the APACHE II and SOFA systems (outcome assessment in Turkish intensive care units). Arch Med Sci. 2018;14(3):510–5. 10.5114/aoms.2016.59709. 29765435 10.5114/aoms.2016.59709 PMC5949908

[96] Jöbges S, Vinay R, Luyckx VA, Biller-Andorno N. Recommendations on COVID-19 triage: international comparison and ethical analysis. Bioethics. 2020;34(9):948–59. 10.1111/bioe.12805. 32975826 10.1111/bioe.12805 PMC7537413

[97] Adams JG, Walls RM. Supporting the health care workforce during the COVID-19 global epidemic. JAMA. 2020;323(15):1439–40. 32163102 10.1001/jama.2020.3972

[98] Aulisio MP, May T. Why healthcare workers ought to be prioritized in ASMR during the SARS-CoV-2 pandemic. Am J Bioeth. 2020;20(7):125–8. 10.1080/15265161.2020.1779411. 32716809 10.1080/15265161.2020.1779411

[99] Cox CL. “Healthcare Heroes”: problems with media focus on heroism from healthcare workers during the COVID-19 pandemic. J Med Ethics. 2020;46(8):510–3. 10.1136/medethics-2020-106398. 32546658 10.1136/medethics-2020-106398 PMC7316119

[100] McGuire AL, Aulisio MP, Davis FD, Erwin C, Harter TD, Jagsi R, Klitzman R, Macauley R, Racine E, Wolf SM, Wynia M, Wolpe PR. COVID-19 task force of the association of bioethics program directors (ABPD) ethical challenges arising in the COVID-19 pandemic: an overview from the association of bioethics program directors (ABPD) task force. Am J Bioeth. 2020. 10.1080/15265161.2020.1764138. 32511078 10.1080/15265161.2020.1764138

[101] de RealAsua D, Fins JJ. Should healthcare workers be prioritised during the COVID-19 pandemic? A view From Madrid. J Med Ethics. 2022. 10.1136/medethics-2020-107050. 10.1136/medethics-2020-107050 33910974

[102] Sveen W, Antommaria AHM. Why healthcare workers should not be prioritized in ventilator triage. Am J Bioeth. 2020;20(7):133–5. 32716811 10.1080/15265161.2020.1779852

[103] Cheung AT, Parent B. Mistrust and inconsistency during COVID-19: considerations for resource allocation guidelines that prioritise healthcare workers. J Med Ethics. 2021;47(2):73–7. 33106381 10.1136/medethics-2020-106801

[104] Kirkpatrick J, Hull S, Fedson S, Mullen B, Goodlin S. Scarce-resource allocation and patient triage during the COVID-19 pandemic. JACC RevTopic Week. 2020;76:85–92. 10.1016/j.jacc.2020.05.006 PMC7213960 32407772

[105] Galiatsatos P, Kachalia A, Belcher HME, Hughes MT, Kahn J, Rushton CH, Suarez JI, Biddison LD, Golden SH. xsHealth equity and distributive justice considerations in critical care resource allocation. Lancet Respir Med. 2020. 10.1016/S2213-2600(20)30277-0. 32585137 10.1016/S2213-2600(20)30277-0 PMC7313886

[106] WHO Headquarters (HQ). Smoking and COVID-19. Scientific brief. 30 June 2020. (Consulted 08 October 2022). WHO/2019-nCoV/Sci_Brief/Smoking/2020.2. https://www.who.int/publications/i/item/WHO-2019-nCoV-Sci_Brief-Smoking-2020.2.

[107] Pinho M, Pinto BA. The views of health care professionals and laypersons concerning the relevance of health-related behaviors in prioritizing patients. Health Educ Behav. 2019;46(5):728–36. 10.1177/1090198119853604. 31204850 10.1177/1090198119853604

[108] Rogge J, Kittel B. Who shall not be treated: public attitudes on setting health care priorities by person-based criteria in 28 nations? PLoS ONE. 2016;11(6):e0157018. 10.1371/journal.pone.0157018. 27280775 10.1371/journal.pone.0157018 PMC4900563

[109] Savulescu J, Persson I, Wilkinson D. Utilitarianism and the pandemic. Bioethics. 2020;34:620–32. 10.1111/bioe.12771. 32433782 10.1111/bioe.12771 PMC7276855

[110] Shaw D. Vaccination status and intensive care unit triage: Is it fair to give unvaccinated COVID-19 patients equal priority? Bioethics. 2022;36:883–90. 10.1111/bioe.13069. 35934875 10.1111/bioe.13069 PMC9538027

[111] Schuman O, Robertson-Preidler J, Bibler TM. COVID-19 vaccination status should not be used in triage tie-breaking. J Med Ethics. 2022. 10.1136/medethics-2021-107836. 34992085 10.1136/medethics-2021-107836

[112] Bieber F. Is nationalism on the rise? Assess Global Trends Ethnopol. 2018;17(5):519–40. 10.1080/17449057.2018.1532633.

[113] UNESCO experts urge collective responsibility to protect vulnerable persons in global battle against COVID-19. Press release 07042020. (Consulted 08 October 2022). https://en.unesco.org/news/unesco-experts-urge-collective-responsibility-protect-vulnerable-persons-global-battle-against.

[114] White DB, Lo B. Mitigating inequities and saving lives with Icu triage during the COVID-19 pandemic. Am J Respir Crit Care Med. 2021;203(3):287–95. 10.1164/rccm.202010-3809CP. 33522881 10.1164/rccm.202010-3809CP PMC7874325

[115] Kirby T. Evidence mounts on the disproportionate effect of COVID-19 on ethnic minorities. Lancet Respir Med. 2020;8(6):547–8. 10.1016/S2213-2600(20)30228-9. 32401711 10.1016/S2213-2600(20)30228-9 PMC7211498

[116] Lauderdale DS, Wen M, Jacobs EA, Kandula NR. Immigrant perceptions of discrimination in health care: the California health interview survey 2003. Med Care. 2006;44(10):914–20. 10.1097/01.mlr.0000220829.87073.f7. 17001262 10.1097/01.mlr.0000220829.87073.f7

[117] Greenaway C, Hargreaves S, Barkati S, Coyle CM, Gobbi F, Veizis A, Douglas P. COVID-19: exposing and addressing health disparities among ethnic minorities and migrants. J Travel Med. 2020. 10.1093/jtm/taaa113. 32706375 10.1093/jtm/taaa113 PMC7454797

[118] Farmer Y. La distribution aléatoire des ressources en santé : pour un modèle hybride équilibrant les principes de justice et de maximisation. Can J of Publ Health/Revue Canadienne de Santé Publique. 2012;103:119–21. 10.1007/BF03404214 PMC6973744 22530533

[119] Cook T, Gupta K, Dyer C, et al. Development of a structured process for fair allocation of critical care resources in the setting of insufficient capacity: a discussion paper. J Med Ethics. 2021;47:456–63. 10.1136/medethics-2021-107361. 10.1136/medethics-2020-106771 PMC7681792 33219013

[120] Silva D. Ventilators by lottery the least unjust form of allocation in the coronavirus disease, pandemic general interest commentary and announcement. CHEST. 2019. 10.1016/j.chest.2020.04.049. 32413344 10.1016/j.chest.2020.04.049 PMC7217110

[121] Wang X. The fairness of ventilator allocation during the COVID-19 pandemic. Bioethics. 2021. 10.1111/bioe.12955. 34536303 10.1111/bioe.12955 PMC8652696

[122] Kanter R. would triage predictors perform better than first-come, first-served in pandemic ventilator allocation? Chest. 2015;147(1):102–8. 25079506 10.1378/chest.14-0564

[123] Apriceno M, Lytle A, Monahan C, Macdonald J, Levy SR. Prioritizing health care and employment resources during COVID-19: roles of benevolent and hostile ageism. Gerontologist. 2021;61(1):98–102. 10.1093/geront/gnaa165. 33119089 10.1093/geront/gnaa165 PMC7665451

[124] Boreskie KF, Boreskie PE, Melady D. Age is just a number - and so is frailty: strategies to inform resource allocation during the COVID-19 pandemic. CJEM. 2020;22(4):411–3. 10.1017/cem.2020.358. 32234101 10.1017/cem.2020.358 PMC7160160

[125] Solomon MZ, Wynia MK, Gostin LO. Covid-19 crisis triage—optimizing health outcomes and disability rights. N Engl J Med. 2020;383(5): e27. 32427434 10.1056/NEJMp2008300

[126] Panocchia N, D’ambrosio V, Corti S, et al. COVID-19 pandemic, the scarcity of medical resources, community-centred medicine and discrimination against persons with disabilities. J Med Ethics. 2021. 10.1136/medethics-2020-107198. 33827907 10.1136/medethics-2020-107198

[127] Scully JL. Disability, disablism, and COVID-19 pandemic triage. Bioeth Inq. 2020;17:601–5. 10.1007/s11673-020-10005-y. 10.1007/s11673-020-10005-y PMC7445721 32840832

[128] Mackenzie C, Scully JL. Moral imagination, disability and embodiment. J Appl Philos. 2007;24:335–51. 10.1111/j.1468-5930.2007.00388.x.

