# Supplementary material for: Public voices on tie-breaking criteria and underlying values in COVID-19 triage protocols to access critical care: a scoping review
Source: Discov Health Syst. 2023 May 10;2(1):16. doi: 10.1007/s44250-023-00027-9 (PMC10169297; doi:10.1007/s44250-023-00027-9)
Supplement: Supplementary file 1 — Supplementary file1 (DOCX 690 KB) [file 44250_2023_27_MOESM1_ESM.docx]

**Additional File 1.** Database Search Strategies

**Ovid MEDLINE**

| Database: Ovid MEDLINE(R) ALL <1946 to February 15, 2022>  Search Strategy:  --------------------------------------------------------------------------------  1 exp COVID-19/ (140402)  2 (Covid-19 or "SARS CoV-2" or pandemic* or "coronavirus disease 2019" or "novel coronavirus" or pandemic*).ab,hw,kf,ot,ti. (258564)  3 *Health Care Rationing/ (6735)  4 *Triage/ (7414)  5 Age Factors/ (470687)  6 COVID-19 Vaccines/ (8972)  7 Smokers/ (3578)  8 healthy lifestyle/ or sedentary behavior/ (14949)  9 survival analysis/ (144121)  10 ("tie-breaker*" or "tie breaker*" or "tie-breaking criteria*" or "tie breaking criteria*" or prioritization or triage or "resource allocation").ab,hw,kf,ot,ti. (56742)  11 (Randomisat* or "random draw*" or "draw lot*" or "drawing lot*" or "draw of lot*" or "drawing of lot*" or "life-cycle*" or "life cycle*" or "first come first served" or "first-come-first-served" or "first-come first-served" or "first come, first served" or "first-come, first-served" or "age" or vaccinated or vaccine* or smok* or "life habit*" or "pre-existing medical condition*" or "pre-existing condition" or "medical condition*" or "chances of surviv*" or "chances for surviv*" or "survival chance*" or "survival analysis").ab,hw,kf,ot,ti. (10009647)  12 ethics consultation/ (1296)  13 Public Opinion/ (19437)  14 focus groups/ or interviews as topic/ or "surveys and questionnaires"/ (605092)  15 ("public consultation*" or "public comment*" or "consult the population" or "consult the public" or "consulting the population" or "consulting the public" or "popular consultation*" or "consultative process*" or "ethics consultation*").ab,hw,kf,ot,ti. (3059)  16 (Survey* or poll* or questionnaire* or "focus group*" or "discussion group*" or "discussion panel*" or "panel discussion*" or interview* or "written consultation*" or "citizens jur*" or "citizens panel*" or "planning cell*" or "consensus conference*" or "deliberative poll*" or "citizen advisory committee*" or referenda* or "public hearing*").ab,hw,kf,ot,ti. (2177082)  17 1 or 2 (258564)  18 3 or 4 or 10 or "28".mp. or "29".mp. [mp=title, abstract, original title, name of substance word, subject heading word, floating sub-heading word, keyword heading word, organism supplementary concept word, protocol supplementary concept word, rare disease supplementary concept word, unique identifier, synonyms] (1140302)  19 5 or 6 or 7 or 8 or 9 or 11 (10012801)  20 12 or 13 or 15 (22423)  21 14 or 16 (2177082)  22 17 and 18 and 19 and 20 and 21 (20)  23 18 and 19 and 20 and 21 (269)  24 limit 23 to (yr="2019 -Current" and (english or french or spanish)) (37)  25 ("tie-breaker*" or "tie breaker*" or "tie-breaking criteria*" or "tie breaking criteria*").ab,hw,kf,ot,ti. (44)  26 limit 25 to ((english or french or spanish) and covid-19) (6)  27 24 or 26 (43)  28 *Resource Allocation/ (4007)  29 *Health Equity/ (1982) |
| --- |

**Embase**

| Database: Embase <1974 to 2022 February 15>  Search Strategy:  --------------------------------------------------------------------------------  1 exp coronavirus disease 2019/ (187446)  2 (Covid-19 or "SARS CoV-2" or pandemic* or "coronavirus disease 2019" or "novel coronavirus" or pandemic*).ab,hw,kf,ot,ti. (285719)  3 *emergency medical dispatch/ (130)  4 *resource allocation/ (4410)  5 *health equity/ (2734)  6 ("tie-breaker*" or "tie breaker*" or "tie-breaking criteria*" or "tie breaking criteria*" or prioritization or triage or "resource allocation").ab,hw,kf,ot,ti. (77014)  7 life cycle/ (21989)  8 vaccinee/ (512)  9 smoking/ or cigarette smoking/ or smoking habit/ (408662)  10 healthy lifestyle/ or sedentary lifestyle/ (23387)  11 disease severity/ (638631)  12 long term survival/ or survival prediction/ or survival analysis/ (74356)  13 (Randomisat* or "random draw*" or "draw lot*" or "drawing lot*" or "draw of lot*" or "drawing of lot*" or "life-cycle*" or "life cycle*" or "first come first served" or "first-come-first-served" or "first-come first-served" or "first come, first served" or "first-come, first-served" or smok* or "life habit*" or "pre-existing medical condition*" or "pre-existing condition" or "medical condition*" or "chances of surviv*" or "chances for surviv*" or "survival chance*" or "survival analysis").ab,hw,kf,ot,ti. (791447)  14 public opinion/ (18242)  15 crowdsourcing/ or interview/ or questionnaire/ (983520)  16 panel study/ (1381)  17 (Survey* or poll* or questionnaire* or "focus group*" or "discussion group*" or "discussion panel*" or "panel discussion*" or interview* or "written consultation*" or "citizens jur*" or "citizens panel*" or "planning cell*" or "consensus conference*" or "deliberative poll*" or "citizen advisory committee*" or referenda* or "public hearing*").ab,hw,kf,ot,ti. (3277479)  18 1 or 2 (285872)  19 3 or 4 or 5 or 6 (79776)  20 7 or 9 or 10 or 11 or 12 or 13 (1459471)  21 14 or 15 or 16 or 17 (3292090)  22 18 and 19 and 20 and 21 (52)  23 19 and 20 and 21 (821)  24 limit 23 to covid-19 (45)  25 22 or 24 (52)  26 limit 25 to ((english or french or spanish) and yr="2019 -Current") (46)  27 limit 26 to embase (40) |
| --- |

**EBM Reviews**

| Database: EBM Reviews - Cochrane Database of Systematic Reviews <2005 to March 30, 2022>, EBM Reviews - ACP Journal Club <1991 to March 2022>, EBM Reviews - Database of Abstracts of Reviews of Effects <1st Quarter 2016>, EBM Reviews - Cochrane Clinical Answers <March 2022>, EBM Reviews - Cochrane Central Register of Controlled Trials <January 2022>, EBM Reviews - Cochrane Methodology Register <3rd Quarter 2012>, EBM Reviews - Health Technology Assessment <4th Quarter 2016>, EBM Reviews - NHS Economic Evaluation Database <1st Quarter 2016>  Search Strategy:  --------------------------------------------------------------------------------  1 (Covid-19 or "SARS CoV-2" or pandemic* or "coronavirus disease 2019" or "novel coronavirus" or pandemic*).ab,hw,kw,ot,sh,ti. (10669)  2 ("tie-breaker*" or "tie breaker*" or "tie-breaking criteria*" or "tie breaking criteria*" or prioritization or triage or "resource allocation").ab,hw,kw,ot,sh,ti. (3077)  3 (Randomisat* or "random draw*" or "draw lot*" or "drawing lot*" or "draw of lot*" or "drawing of lot*" or "life-cycle*" or "life cycle*" or "first come first served" or "first-come-first-served" or "first-come first-served" or "first come, first served" or "first-come, first-served" or "age" or vaccinated or vaccine* or smok* or "life habit*" or "pre-existing medical condition*" or "pre-existing condition" or "medical condition*" or "chances of surviv*" or "chances for surviv*" or "survival chance*" or "survival analysis").ab,hw,kw,ot,sh,ti. (442998)  4 ("Health care rationing" or triage or "resource allocation" or "health equity").ab,hw,kw,ot,sh,ti. (2943)  5 (Age factors or COVID-19 vaccines or Smokers or "healthy lifestyle" or "sedentary behavior" or "survival analysis").ab,hw,kw,ot,sh,ti. (44191)  6 ("public consultation*" or "public comment*" or "consult the population" or "consult the public" or "consulting the population" or "consulting the public" or "popular consultation*" or "consultative process*" or "ethics consultation*").ab,hw,kw,ot,sh,ti. (60)  7 (Survey* or poll* or questionnaire* or "focus group*" or "discussion group*" or "discussion panel*" or "panel discussion*" or interview* or "written consultation*" or "citizens jur*" or "citizens panel*" or "planning cell*" or "consensus conference*" or "deliberative poll*" or "citizen advisory committee*" or referenda* or "public hearing*").ab,hw,kw,ot,sh,ti. (226694)  8 ("Ethics consultation" or "public opinion").ab,hw,kw,ot,sh,ti. (255)  9 2 or 4 (3303)  10 3 or 5 (445765)  11 6 or 8 (297)  12 1 and 7 and 9 and 10 (10)  13 6 or 7 or 8 (226840)  14 1 and 9 and 10 and 13 (10) |
| --- |

**APA PsycInfo**

| Database: APA PsycInfo <1806 to March Week 3 2022>  Search Strategy:  --------------------------------------------------------------------------------  1 (Covid-19 or "SARS CoV-2" or pandemic* or "coronavirus disease 2019" or "novel coronavirus" or pandemic*).ab,hw,ot,ti. (17311)  2 exp COVID-19/ (8009)  3 ("tie-breaker*" or "tie breaker*" or "tie-breaking criteria*" or "tie breaking criteria*" or prioritization or triage or "resource allocation").ab,hw,ot,ti. (10242)  4 (Randomisat* or "random draw*" or "draw lot*" or "drawing lot*" or "draw of lot*" or "drawing of lot*" or "life-cycle*" or "life cycle*" or "first come first served" or "first-come-first-served" or "first-come first-served" or "first come, first served" or "first-come, first-served" or "age" or vaccinated or vaccine* or smok* or "life habit*" or "pre-existing medical condition*" or "pre-existing condition" or "medical condition*" or "chances of surviv*" or "chances for surviv*" or "survival chance*" or "survival analysis").ab,hw,ot,ti. (661215)  5 *Resource Allocation/ (2516)  6 *health disparities/ (7269)  7 exp life span/ (11786)  8 exp chronological age/ or exp age discrimination/ (1461)  9 exp Tobacco Smoking/ (35849)  10 exp active living/ or exp health behavior/ (39358)  11 exp life expectancy/ (3655)  12 ("public consultation*" or "public comment*" or "consult the population" or "consult the public" or "consulting the population" or "consulting the public" or "popular consultation*" or "consultative process*" or "ethics consultation*").ab,hw,ot,ti. (531)  13 (Survey* or poll* or questionnaire* or "focus group*" or "discussion group*" or "discussion panel*" or "panel discussion*" or interview* or "written consultation*" or "citizens jur*" or "citizens panel*" or "planning cell*" or "consensus conference*" or "deliberative poll*" or "citizen advisory committee*" or referenda* or "public hearing*").ab,hw,ot,ti. (891940)  14 exp public opinion/ (9277)  15 interviews/ or questionnaires/ or surveys/ (45626)  16 focus group/ or narrative analysis/ or semi-structured interview/ (3034)  17 crowdsourcing/ (704)  18 1 or 2 (17311)  19 3 or 5 or 6 (17464)  20 4 or 7 or 8 or 9 or 10 or 11 (698055)  21 12 or 14 (9781)  22 13 or 15 or 16 or 17 (893099)  23 19 and 20 (3374)  24 21 and 22 (4422)  25 18 and 23 and 24 (2)  26 21 or 22 (898458)  27 18 and 23 and 26 (18) |
| --- |

**PubMed**

| **On:** Fri Apr 08 17:42:28 2022  **PubMed: Third search**  **Search:** ((Covid-19[Title/Abstract] OR "SARS CoV-2"[Title/Abstract] OR pandemic*[Title/Abstract] OR "coronavirus disease 2019"[Title/Abstract] OR "novel coronavirus"[Title/Abstract]) OR (COVID-19[MeSH Terms])) AND ((("tie-breaker*"[Title/Abstract] OR "tie breaker*"[Title/Abstract] OR "tie-breaking criteria*"[Title/Abstract] OR "tie breaking criteria*"[Title/Abstract] OR prioritization[Title/Abstract] OR triage[Title/Abstract] OR "resource allocation"[Title/Abstract]) OR (Health care rationing OR triage OR resource allocation OR health equity[MeSH Terms])) OR ((Randomisat*[Title/Abstract] OR "random draw*"[Title/Abstract] OR "draw lot*"[Title/Abstract] OR "drawing lot*"[Title/Abstract] OR "draw of lot*"[Title/Abstract] OR "drawing of lot*"[Title/Abstract] OR "life-cycle*"[Title/Abstract] OR "life cycle*"[Title/Abstract] OR "first come first served"[Title/Abstract] OR "first-come-first-served"[Title/Abstract] OR "first-come first-served"[Title/Abstract] OR "first come, first served"[Title/Abstract] OR "first-come, first-served"[Title/Abstract] OR "age"[Title/Abstract] OR "pre-existing medical condition*"[Title/Abstract] OR "pre-existing condition"[Title/Abstract] OR "medical condition*"[Title/Abstract] OR "chances of surviv*"[Title/Abstract] OR "chances for surviv*"[Title/Abstract] OR "survival chance*"[Title/Abstract] OR "survival analysis"[Title/Abstract]) OR (Age factors OR survival analysis[MeSH Terms]))) AND (("public consultation*"[Title/Abstract] OR "public comment*"[Title/Abstract] OR "consult the population"[Title/Abstract] OR "consult the public"[Title/Abstract] OR "consulting the population"[Title/Abstract] OR "consulting the public"[Title/Abstract] OR "popular consultation*"[Title/Abstract] OR "consultative process*"[Title/Abstract] OR "ethics consultation*"[Title/Abstract]) OR (Ethics consultation OR public opinion[MeSH Terms])) AND ((Survey*[Title/Abstract] OR poll*[Title/Abstract] OR questionnaire*[Title/Abstract] OR "focus group*"[Title/Abstract] OR "discussion group*"[Title/Abstract] OR "discussion panel*"[Title/Abstract] OR "panel discussion*"[Title/Abstract] OR interview*[Title/Abstract] OR "written consultation*"[Title/Abstract] OR "citizens jur*"[Title/Abstract] OR "citizens panel*"[Title/Abstract] OR "planning cell*"[Title/Abstract] OR "consensus conference*"[Title/Abstract] OR "deliberative poll*"[Title/Abstract] OR "citizen advisory committee*"[Title/Abstract] OR referenda*[Title/Abstract] OR "public hearing*"[Title/Abstract]) OR (focus groups OR interviews as topic OR surveys and questionnaires[MeSH Terms])) Filters: from 2020 - 2022  51 selected items |
| --- |

**CINAHL**

**March 2022. Results: 58 records**


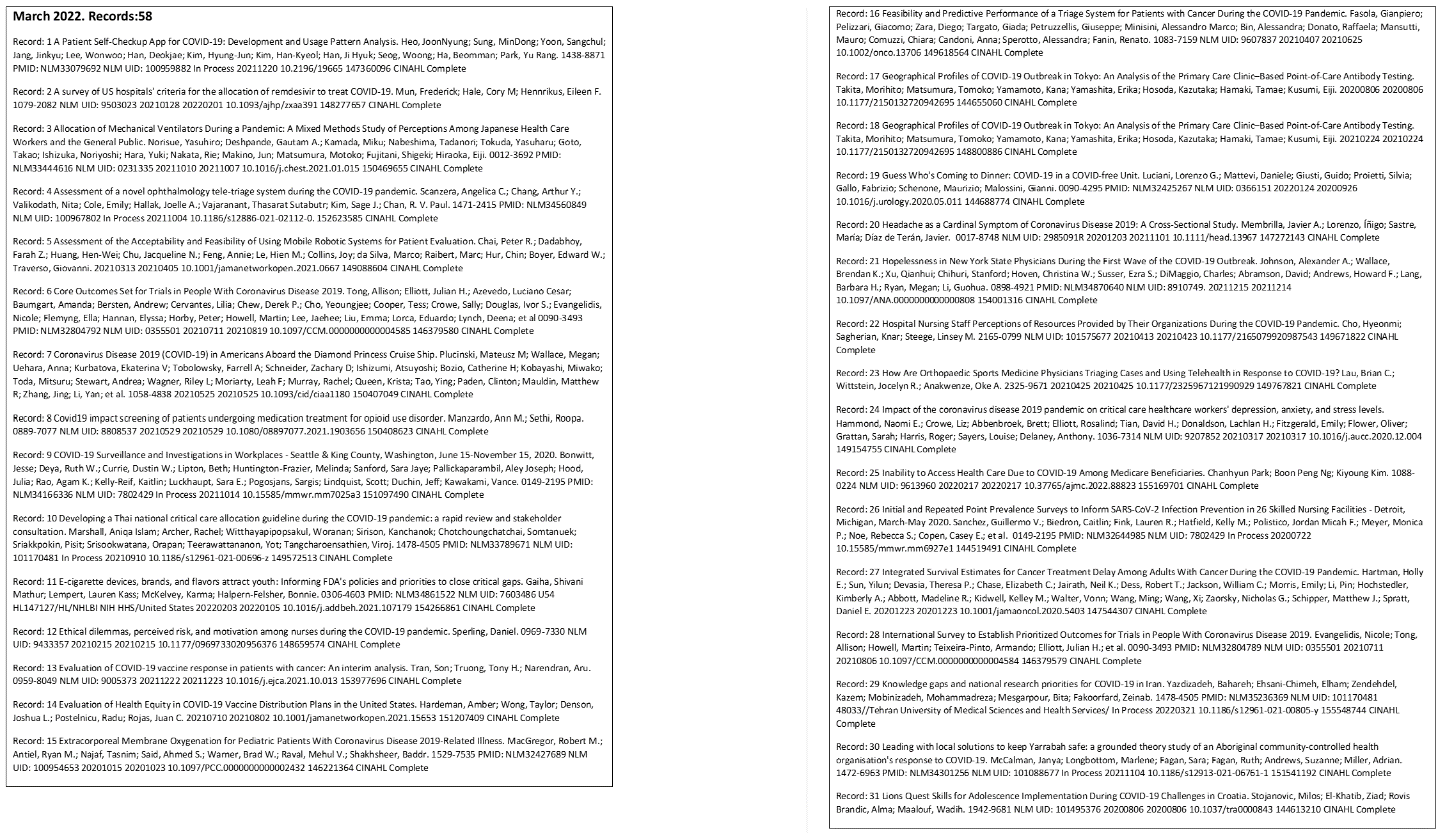


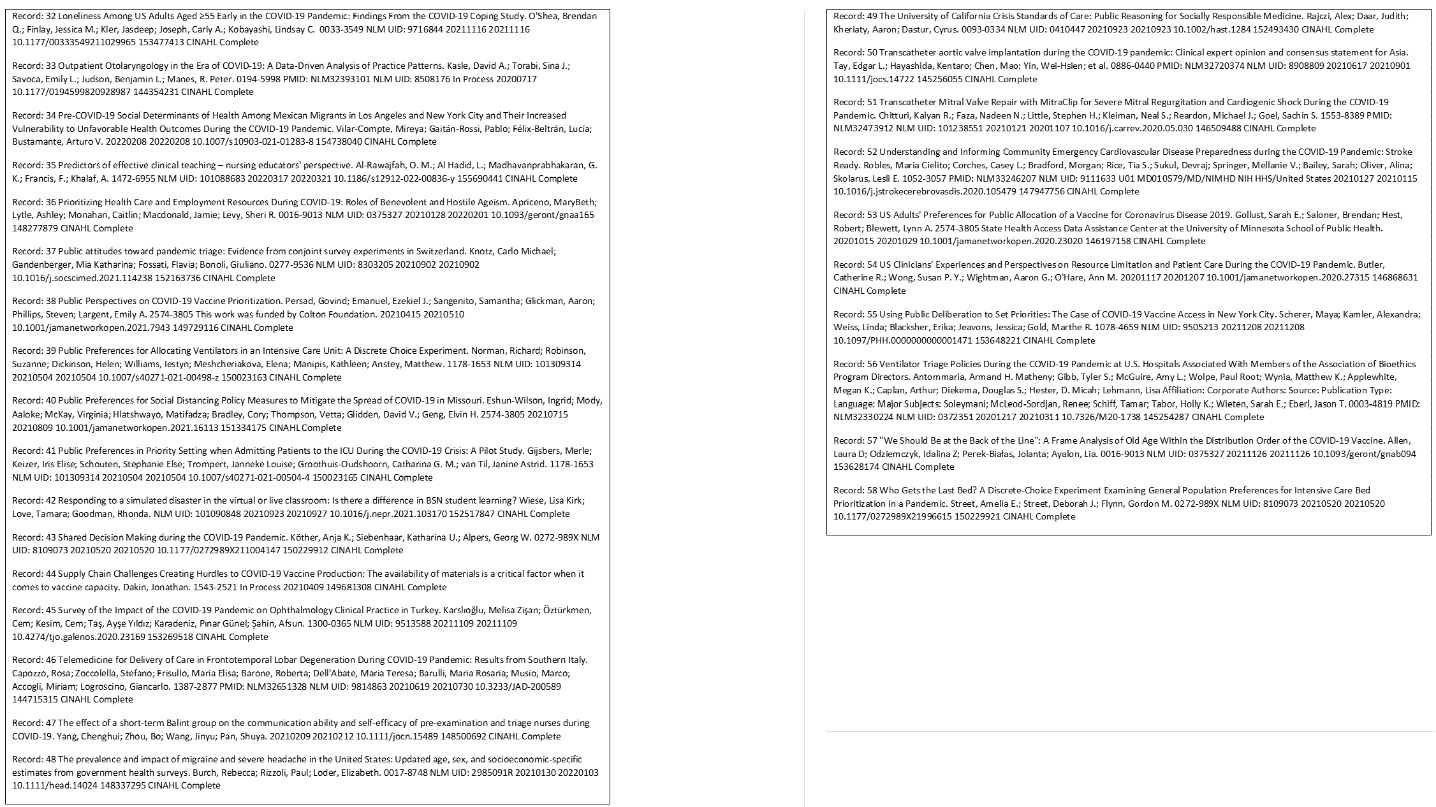


**Web of Science**

**April 2022. Results sheet 1**

| 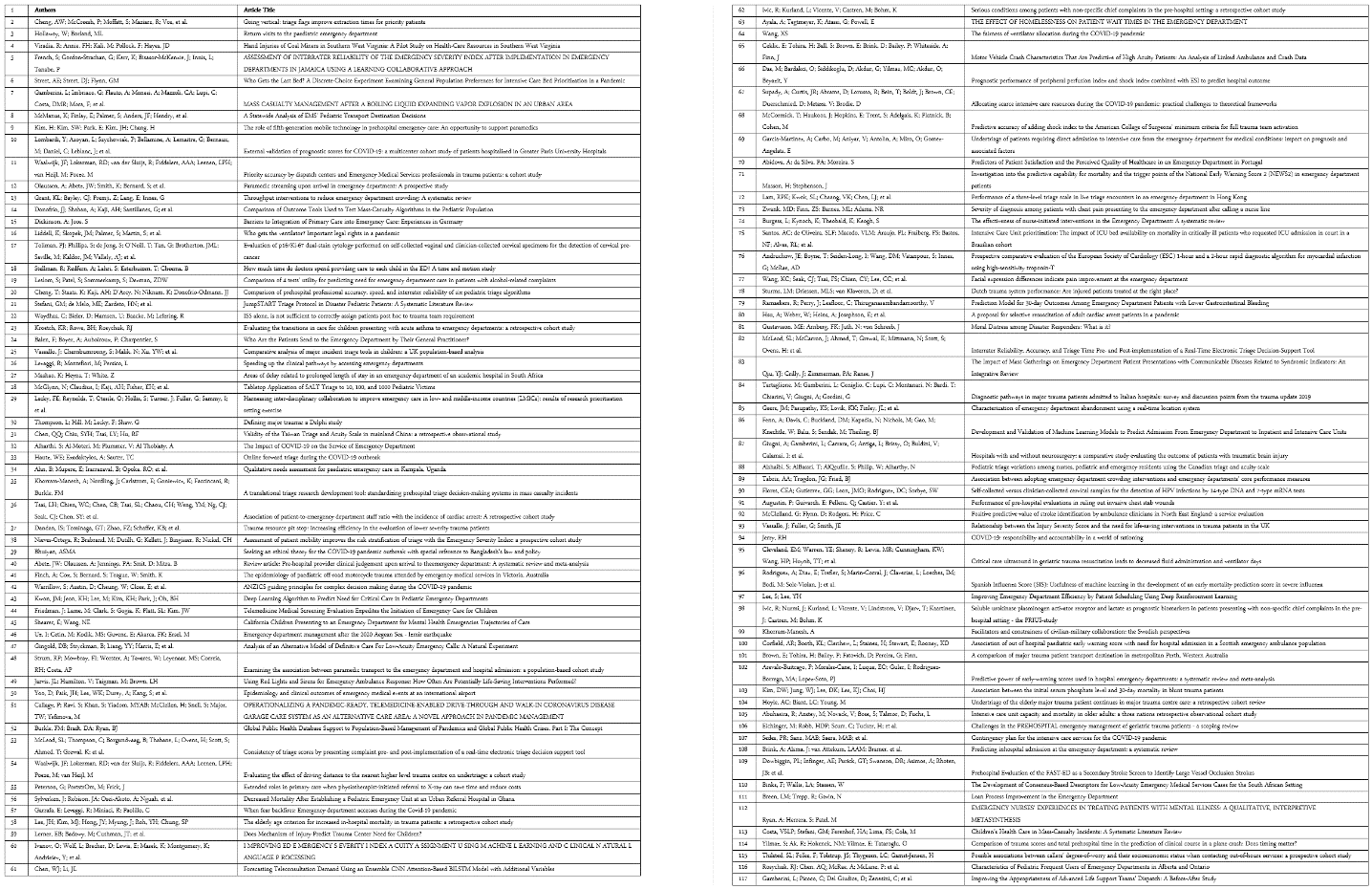 |
| --- |

**April 2022. Results sheet 2**

| 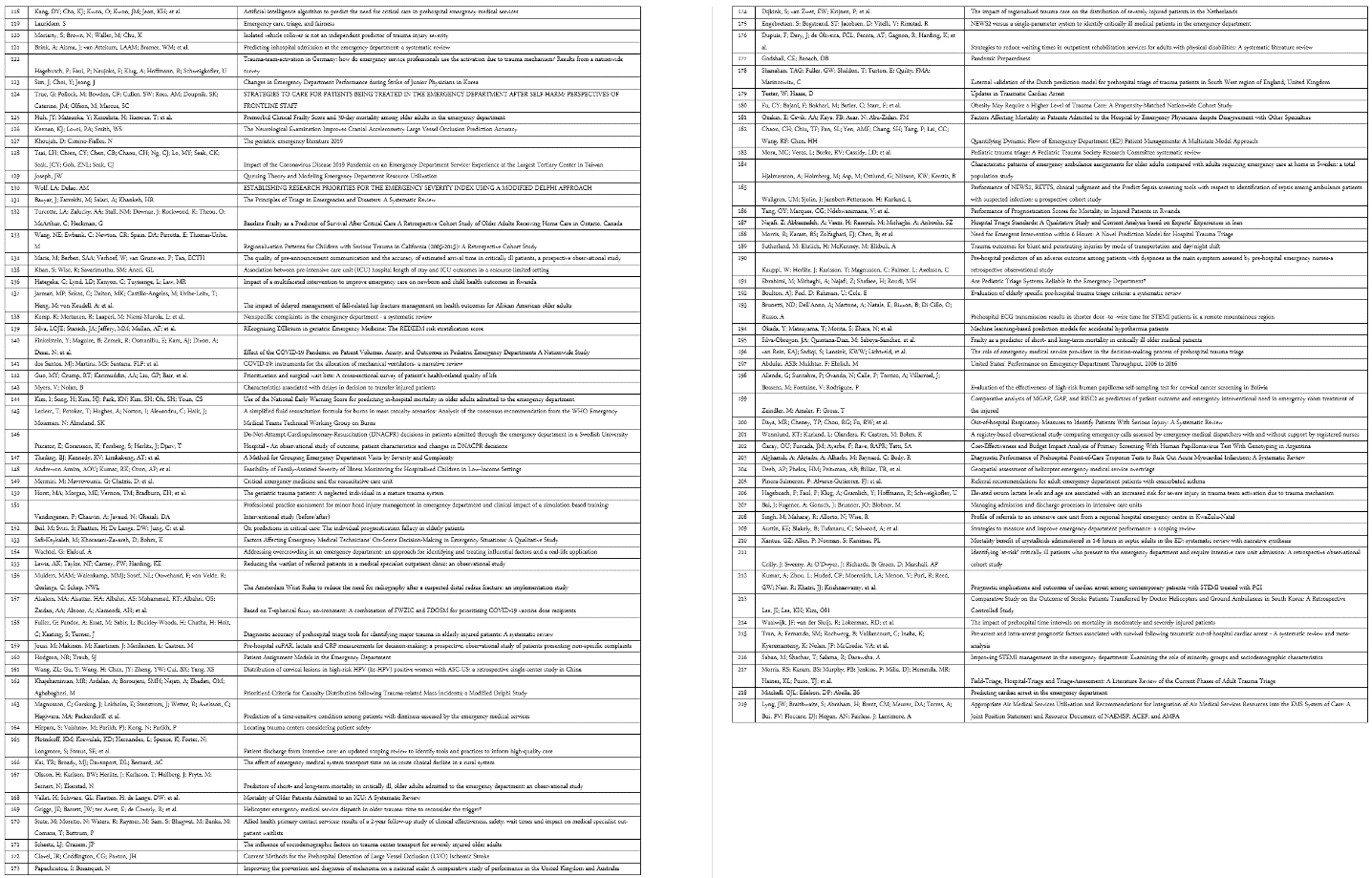 |
| --- |

| **Scoping review. Résumé des concepts clés**  **Concept 1 – Covid-19**  (Concept remplacé par la limite covid-19 lorsque disponible, en surplus de la recherche avec le concept)  Vocabulaire libre  Covid-19 OR "SARS CoV-2" OR pandemic* OR "coronavirus disease 2019" OR "novel coronavirus" OR pandemic*  Vocabulaire contrôlé  Medline : COVID-19 (explode) Embase : coronavirus disease 2019 (explode) CINAHL  PsycInfo  Web of science  EBM reviews - Cochrane   **Concept 2 : Critères de priorisation**  Vocabulaire libre  "tie-breaker*" OR "tie breaker*" OR "tie-breaking criteria*" OR "tie breaking criteria*" OR prioritization OR triage OR "resource allocation"  AND  Randomisat* OR "random draw*" OR "draw lot*" OR "drawing lot*" OR "draw of lot*" OR "drawing of lot*" OR "life-cycle*" OR "life cycle*" OR "first come first served" OR "first-come-first-served" OR "first-come first-served" OR "first come, first served" OR "first-come, first-served" OR "age" OR vaccinated OR vaccine* OR smok* OR "life habit*" OR "pre-existing medical condition*" OR "pre-existing condition" OR "medical condition*" OR "chances of surviv*" OR "chances for surviv*" OR "survival chance*" OR "survival analysis"  Vocabulaire contrôlé  Medline: Health care rationing (focus) OR triage (focus) OR resource allocation (focus) OR   health equity (focus)  AND Age factors OR COVID-19 vaccines OR Smokers OR healthy lifestyle OR sedentary   behavior OR survival analysis  Embase: emergency medical dispatch (focus) OR resource allocation (focus) OR health equity   (focus)  AND  life cycle OR age OR ~~vaccinee~~ OR smoking OR cigarette smoking OR smoking habit OR   healthy lifestyle OR sedentary lifestyle OR disease severity OR long term survival OR   survival analysis OR survival prediction CINAHL  PsycInfo  Web of science  EBM reviews - Cochrane   **Concept 3 : Consultations publiques**  Vocabulaire libre  "public consultation*" OR "public comment*" OR "consult the population" OR "consult the public" OR "consulting the population" OR "consulting the public" OR "popular consultation*" OR "consultative process*" OR "ethics consultation*"  AND (ou OR, selon la base de données)  Survey* OR poll* OR questionnaire* OR "focus group*" OR "discussion group*" OR "discussion panel*" OR "panel discussion*" OR interview* OR "written consultation*" OR "citizens jur*" OR "citizens panel*" OR "planning cell*" OR "consensus conference*" OR "deliberative poll*" OR "citizen advisory committee*" OR referenda* OR "public hearing*"  Vocabulaire contrôlé  Medline: Ethics consultation OR public opinion  AND focus groups OR interviews as topic OR surveys and questionnaires Embase:  public opinion OR crowdsourcing OR interview OR questionnaire OR panel study  CINAHL  PsycInfo  Web of science  EBM reviews - Cochrane |
| --- |
