# Supplementary material for: Public voices on tie-breaking criteria and underlying values in COVID-19 triage protocols to access critical care: a scoping review
Source: Discov Health Syst. 2023 May 10;2(1):16. doi: 10.1007/s44250-023-00027-9 (PMC10169297; doi:10.1007/s44250-023-00027-9)
Supplement: Supplementary file 2 — Supplementary file2 (DOCX 18 KB) [file 44250_2023_27_MOESM2_ESM.docx]

**Additional File 2.** Synthesis of thematic analysis

| **Themes** | **Sub-themes** | **Values considered by consulted public** | **References** |
| --- | --- | --- | --- |
| **I. An indirect and direct approach based on patient age** | 1. Intergenerational equity  2. Absolute age  3. The “saving more years of life” principle | “Fair innings”/Equity  Patient age  Expectancy of life | [40, 44, 46, 51, 52, 55, 56, 58, 59]  [40, 41, 48, 49, 52, 53, 56, 59]  [45, 57] |
| **II. The social and instrumental value** | 1. Essential healthcare personnel  2. Essential non-healthcare people  3. Merit as a value  a) Individual and collective health behavior  b) In healthcare workers or first responders  c) In criminals  4. Nationality as a value | Reciprocity/Solidarity  Multiplier effect  Instrumental/Solidarity  Patient merit  Patient nationality | [41, 42, 46, 48, 50, 58]  [42, 43, 46, 48, 49, 56]  [46, 48, 49, 51, [52, 58]  [42, 43, 54, 55]  [42, 43, 58]  [40, 41, 46]  [40, 49, 54, 59] |
| **III. The egalitarian perspective** | 1. The "first-come, first-served" principle  2. Randomization | Equality | [43, 47, 59]  [48] |
| **IV. Solidarity towards the most vulnerable** | 1. Quality of life  2. Pandemic disease priority | Patient quality of life  COVID-19 patient | [46, 47]  [41, 43] |
| **V. Efficacity and stewardship** | 1. Short-term patient recovery  2. Financial and human cost | Maximizing benefits  Prevention and health economics | [47, 48, 58]  [40] |
